# Emerging algorithmic bias: fairness drift as the next dimension of model maintenance and sustainability

**DOI:** 10.1093/jamia/ocaf039

**Published:** 2025-03-13

**Authors:** Sharon E Davis, Chad Dorn, Daniel J Park, Michael E Matheny

**Affiliations:** Department of Biomedical Informatics, Vanderbilt University Medical Center, Nashville, TN 37203, United States; Department of Biomedical Informatics, Vanderbilt University Medical Center, Nashville, TN 37203, United States; Department of Biomedical Informatics, Vanderbilt University Medical Center, Nashville, TN 37203, United States; Department of Biomedical Informatics, Vanderbilt University Medical Center, Nashville, TN 37203, United States; Department of Medicine, Vanderbilt University Medical Center, Nashville, TN 37232, United States; Department of Biostatistics, Vanderbilt University Medical Center, Nashville, TN 37203, United States; Tennessee Valley Healthcare System VA Medical Center, Veterans Health Administration, Nashville, TN 37212, United States

**Keywords:** algorithmic fairness, dataset shift, predictive analytics, model updating, performance drift

## Abstract

**Objectives:**

While performance drift of clinical prediction models is well-documented, the potential for algorithmic biases to emerge post-deployment has had limited characterization. A better understanding of how temporal model performance may shift across subpopulations is required to incorporate fairness drift into model maintenance strategies.

**Materials and Methods:**

We explore fairness drift in a national population over 11 years, with and without model maintenance aimed at sustaining population-level performance. We trained random forest models predicting 30-day post-surgical readmission, mortality, and pneumonia using 2013 data from US Department of Veterans Affairs facilities. We evaluated performance quarterly from 2014 to 2023 by self-reported race and sex. We estimated discrimination, calibration, and accuracy, and operationalized fairness using metric parity measured as the gap between disadvantaged and advantaged groups.

**Results:**

Our cohort included 1 739 666 surgical cases. We observed fairness drift in both the original and temporally updated models. Model updating had a larger impact on overall performance than fairness gaps. During periods of stable fairness, updating models at the population level increased, decreased, or did not impact fairness gaps. During periods of fairness drift, updating models restored fairness in some cases and exacerbated fairness gaps in others.

**Discussion:**

This exploratory study highlights that algorithmic fairness cannot be assured through one-time assessments during model development. Temporal changes in fairness may take multiple forms and interact with model updating strategies in unanticipated ways.

**Conclusion:**

Equitable and sustainable clinical artificial intelligence deployments will require novel methods to monitor algorithmic fairness, detect emerging bias, and adopt model updates that promote fairness.

## Introduction

Deploying analytic artificial intelligence (AI) models in clinical settings requires careful assessment and detailed understanding of model performance, not only among the population as a whole but also among key subgroups.^1^ Differences in model accuracy between subpopulations—be they demographic, economic, clinical, or geographic—can exacerbate existing health disparities and discriminate in access to care.^2^ Understanding and characterizing algorithmic fairness remains a challenge, with conflicting definitions and perspectives on fairness and bias in clinical decision-making.^2^^,^^3^ However, given the importance of advancing health equity alongside the increasingly rapid deployment of clinical AI solutions, algorithmic fairness has been integrated into best practice recommendations even as novel methods and metrics for equitable AI continue to develop.^2^^,^^4–9^

Responsible deployment of clinical AI further requires that the evaluation of algorithmic fairness extends beyond model development to include post-deployment sustainability.^10^ The tendency of model accuracy to drift over time is a well-documented consequence of temporal changes in clinical practice, patient populations, and information systems.^11–13^ Dramatic drift in the distributions of patient characteristics and symptoms in the early months of the coronavirus pandemic disrupted the performance of a sepsis model, leading to a 43% increase in alerts and the model being temporarily disabled.^14^^,^^15^ More subtle drift has been linked to deteriorating calibration of patient-level predictions^13^^,^^16^^,^^17^ and misleadingly optimistic quality evaluations.^18^^,^^19^ As a result, model monitoring and maintenance at the population level have been incorporated into the AI lifecycle.^20^^,^^21^ The potential for algorithmic biases to emerge post-deployment, however, has yet to be characterized. Fairness drift—either due to differences in dataset shift across subpopulations or the model updating process itself—raises concerns that models that were once fair will begin contributing to health inequities. Evaluations of current methods for model maintenance have not addressed their ability to prevent and correct fairness drift and emerging bias. Novel approaches to model monitoring and updating may be necessary to sustain algorithmic fairness in clinical AI tools.

A better understanding of how model performance may shift over time across subpopulations is required to incorporate fairness drift into model maintenance strategies currently focused on sustaining population-level performance. In this study, we explore fairness drift in a national population using models based on the American College of Surgeons National Surgical Quality Improvement Program (ACS NSQIP) universal risk calculator. For over 10 years, the ACS NSQIP risk calculator has provided patient-level risk estimates for common post-surgical complications to support decision-making and informed consent.^22^ The ACS has long acknowledged the importance of model maintenance with prescheduled semi-annual retraining of their quality benchmarking model^23^ and repeated updating of their public risk calculator, including a recent revision to leverage a new learning algorithm for improved performance.^24^ These efforts, however, have not considered algorithmic fairness to guide the timing or evaluation of updated models.

Using the ACS NSQIP universal risk calculator’s modeling structure, we explore the performance of models predicting mortality, unplanned readmission, and pneumonia within 30 days following a surgical procedure over the course of 11 years in a diverse national population of veterans receiving care across all 50 US states. Acknowledging that some definitions of fairness are inherently in conflict and different use cases may prioritize different perspectives of fairness,^2^ in this exploratory work, we consider metric parity across multiple dimensions of model performance. We also consider whether and how fairness drift is reflected in population-level measures of performance and the influence of guiding model maintenance at the population level.

## Methods

### Study design

The objective of this study was to explore fairness drift and the impact of risk prediction model updating on algorithmic fairness. We selected a clinical domain in which the data collection and outcome definitions were well characterized and in which there are established risk prediction tools, namely the ACS NSQIP universal risk calculator.^22^^,^^24^

**Figure 1.**
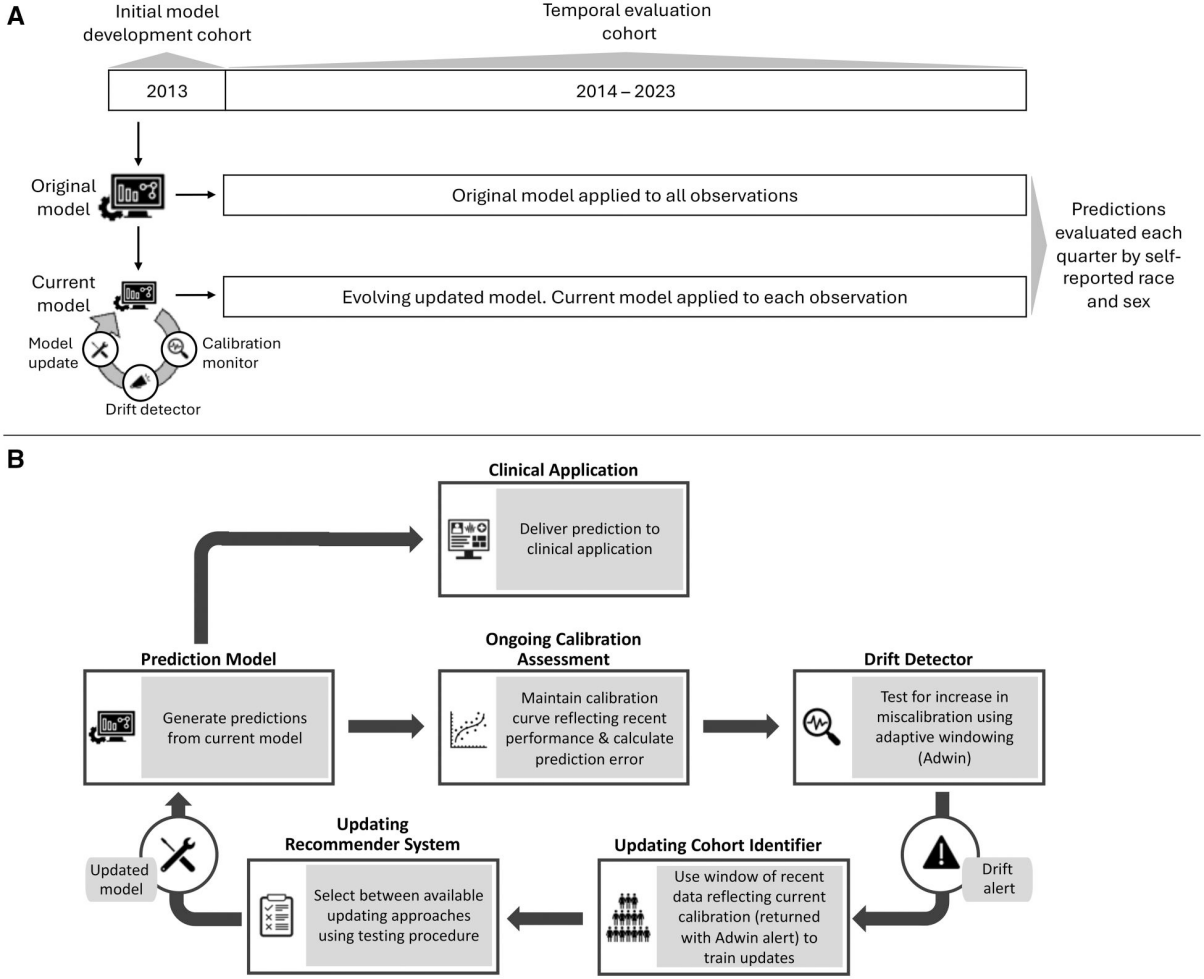
Study design (A) and data-driven surveillance-based model maintenance approach (B). Data from 2013 were used for model training, with data from 2014 to 2023 used for temporal analyses of the original model as well as surveillance and updating of models evolving in response to changing model performance. Model surveillance involved dynamic calibration curves that shift in response to changing model performance; adaptive windowing (Adwin) drift detectors to alert if miscalibration increased and inform subsequent updating process; and select between updating approaches using a nonparametric testing procedures design to promote prospective performance while preferring conservative model adaptations.

We developed and evaluated supervised machine learning risk prediction models for 3 post-surgical adverse events among a national cohort of patients undergoing surgeries performed at US Department of Veterans Affairs (VA) facilities between 2013 and 2023. Original models based on 1 year of data and models updated in response to population-level performance drift were evaluated over 10 years. Model evaluation was conducted quarterly with key discrimination and calibration-related modeling metrics. To assess sub-population performance, algorithmic fairness over time was considered by self-reported race and sex. Figure 1 provides an illustration of the study design and data flow. This study was approved by the institutional review board of the Tennessee Valley Healthcare System VA Medical Center and informed consent was waived due to infeasibility of contact and minimal risk criteria for secondary use of observational data.

### Outcomes

The ACS NSQIP universal risk calculator provides predictions for 13 post-surgical complications.^25^ In this study, we focus on 3 of these outcomes—30-day mortality, unplanned readmission, and pneumonia. These outcomes represent common post-surgical concerns, serious complications, and key quality measures. Briefly, we define mortality as death within 30 days of the surgical procedure whether during inpatient stay or after discharge; unplanned readmission within 30 days of the surgical procedure based on the Centers for Medicare & Medicaid Services definition^26^; and new diagnosis of pneumonia within 30 days of the surgical procedure.

### Cohort inclusion/exclusion

We extracted data on all patients who were undergoing a surgical procedure in which the primary procedure current procedural terminology (CPT) code was included in the ACS NSQIP program.^22^ Surgeries were excluded if the patient was less than 18 years of age or if the primary surgical procedure CPT code occurred in less than 25 procedures within the study period. Surgical procedures were also excluded in the final quarter of 2023 (October-December) to allow outcome follow-up within our study period.

### Data collection and preparation

We accessed data from the VA’s Corporate Data Warehouse, a national repository of VA administrative claims and clinical data extracted from the production electronic health record system. This data resource includes information on over 15 million veterans receiving care since 2000 at VA facilities nationwide. For all eligible cases, we extracted information on demographics, comorbidities, procedures, vitals, and care utilization. Candidate features reflected the definitions in the previously published the ACS NSQIP universal risk calculator^22^ and were based on data collected prior to surgery. Where prior publications provided limited details of feature definitions, we implemented similar features based on available information and existing recommendations. For example, we used the Elixhauser index to define chronic comorbidities and included all Elixhauser conditions, even if only some reflected previously included predictors. In the pneumonia model, we also included the month of surgery as a predictor to account for seasonality. Race and ethnicity were not included as candidate features in the models. A full list of ACS NSQIP features and comparison with features included in our models is provided in Appendix S1.

### Model development and maintenance

We designated surgical cases occurring in 2013 for model training, reserving data from 2014 to 2023 for temporal analyses (see Figure 1A). The 2013 cases were split into 70% training, 10% validation, and 20% test sets. For each outcome—mortality, unplanned readmission, and pneumonia within 30 days—we fit a random forest model (implementation: ranger package v0.16.0 in R 4.4.1) on the training set using the candidate features derived from the ACS NSQIP universal risk calculator as described above. We selected random forest as the learning algorithm as this is a common machine learning approach in clinical prediction studies and inherently incorporates potential non-linearity and interactions within feature relationships. Models for each outcomes were separately tuned for the number of predictors, number of trees, and tree depth using a holdout validation set and Bayes optimization. These initial models were applied to the 2013 test set for initial validation, as well as to all cases in the temporal analysis period (2014-2023) for temporal validation.

We also implemented data-driven surveillance-based model maintenance (see Figure 1B) over the course of the 10-year period following model development (2014-2023). Performance was monitored and models were updated at the population level, reflecting current practice in which maintenance strategies are focused on a common model for the entire patient population. We monitored performance using dynamic calibration curves, triggered updates when deterioration in calibration was identified by a drift detector, and adapted models with recent observations based on the updating method recommended by a nonparametric testing procedure.^27^^,^^28^ Dynamic calibration curves evolve to reflect recent model performance using online stochastic gradient descent to allow logistic calibration curves to shift in response to changes in model performance as data accumulate over time, with the speed of curve evolution regulated using Adam optimization.^27^ We used the adaptive windowing (Adwin) drift detection algorithm to monitor calibration errors over time. Adwin evaluated the temporal series of predictions against up-to-date dynamic calibration curves and tracked trends in miscalibration. We implemented a one-sided Adwin test to only alert when deteriorating model performance (ie, increases in miscalibration) was observed.^27^ In addition to alerting when model calibration deteriorates, Adwin returns a window of recent observations reflecting current model performance patterns. We used this returned data window to train model updates and selected between updating approaches using a testing procedure designed to make conservative updating recommendations that improve performance while minimizing model adaptations.^28^ We allowed the testing procedure to consider retaining the current model, intercept correction, linear recalibration, and flexible recalibration. The timing of performance drift detection and any model updates applied were documented.

All model development, performance monitoring, drift detection, and updating were conducted in R 4.4.1 using publicly available packages. R and Python implementations of dynamic calibration curves and one-sided Adwin are publicly available at https://github.com/sedavis/ModelSurv.

### Modeling evaluation

Initial model performance expectations were established using data in the 2013 test set. We estimated an ensemble of discrimination (area under the receiver operating characteristics curve [AUC]) and calibration (observed to expected outcome ratio [O:E], slope, intercept, integrated calibration index [ICI]) metrics and classification accuracy, positive predicted value (PPV), negative predicted value (NPV), sensitivity, and specificity. Classification thresholds for each model were based on the predicted probability that maximized the mean of the specificity and sensitivity in the entire population. All measures were estimated overall and separately by self-reported race and sex. Due to small sample sizes, race-specific subpopulation analyses compared White and Black patients only. Confidence intervals were estimated through bootstrapping.

Algorithmic fairness was evaluated using metric parity measured as the difference (gap; ideal value = 0) between values estimated in the disadvantaged versus advantaged group.^7^^,^^29^^,^^30^ We note that for some metrics, fairness gaps are to be expected by definition if a common classification threshold is selected when outcome rates differ across subpopulations.^2^ Differences in initial performance across subpopulations were evaluated by comparing bootstrapped 95% confidence intervals of fairness gaps against the null gap of 0. We note that in these analyses, we were primarily interested in temporal changes in fairness gaps rather than whether a gap initially existed after model training.

We conducted temporal monitoring of performance and fairness of the original models trained on 2013 cases and models actively updated with data-driven surveillance. Under each scenario, all model performance and fairness measures were evaluated quarterly over the 10 years from 2014 to 2023, overall and by self-reported race and sex. We compared trends in overall and subpopulation performance metrics, as well as trends in algorithmic fairness metrics. We visually evaluated temporal trends using summary metrics and bootstrapped confidence intervals. In addition, using general linear models, we tested for linear trends in performance and fairness gaps over time for the original models and models subject to surveillance-based updating. We did not control for multiple comparisons due to the exploratory nature of this study; however, we report *P* values using the more rigorous threshold of .01 rather than the common .05 to help alleviate some concern regarding Type 1 errors.

## Results

Our cohort included 1 739 666 surgical cases involving 1 041 947 unique patients, with 172 345 and 1 567 321 cases occurring in the 2013 training period and the 2014-2023 temporal validation period, respectively. Table 1 provides an overview of the study population. Most cases were outpatient surgeries (60%) involving primarily White (79%) and male (91%) patients. We note that the proportion of surgical cases involving female patients has been rising from 7.6% in 2013 to 11.5% in 2023. Post-surgical readmission was observed in 8.6% of cases, with the rate declining from 9.3% in 2013 to 7.3% in 2023. Post-surgical mortality and pneumonia were more consistent over time, with overall rates of 1% and 2%, respectively (see Figure S1). For all 3 outcomes, anomalies in outcome rates were observed in 2020, coinciding with the early phases of the coronavirus pandemic. Outcome rates were lower for female than male patients throughout the study period. Readmission rates were higher for Black vs White patients, particularly between 2016 and 2020.

**Table 1.** Study population of eligible surgical cases occurring in the VA between 2013 and 2023.

	Training period (2013)		Temporal validation period (2014-2023)	
	*N*	%	*N*	%
Encounters	172 345		1 567 321	
Unique patients	136 808		905 139	
Outpatient case	101 079	58.6	954 565	60.9
Age (median [IQR])	66 [55, 69]		63 [57, 72]	
Female	13 118	7.6	145 559	9.3
Race				
Asian/Pacific Islander	1776	1.1	19 688	1.3
Black	29 886	18.4	286 349	19.3
Native American/Alaskan Native	1171	0.7	11 396	0.8
White	129 990	79.8	1 166 705	78.6
Hispanic ethnicity	9790	5.8	98 842	6.4
Tobacco use	67 528	39.2	532 006	33.9
Diabetes	56 866	33.0	548 309	35.0
CHF	17 080	9.9	193 050	12.3
Hypertension with complication	10 215	5.9	164 588	10.5
Renal failure	19 853	11.5	228 932	14.6
BMI (median [IQR])	28.6 [25.1, 32.7]		28.9 [25.3, 33.1]	
Outcomes				
Mortality within 30 days	1858	1.1	15 117	1.0
Readmission within 30 days	16 003	9.3	132 796	8.5
Pneumonia within 30 days	3970	2.3	36 750	2.3

Initial population-level performance of each model on the full test set from 2013 is provided in Table 2. Classification thresholds that maximized the mean of the specificity and sensitivity in the entire population were 3.8% for mortality, 8.8% for readmission, and 3.7% for pneumonia. Discrimination was generally high and followed patterns previously documented for the NSQIP universal risk calculator.^25^ The readmission model had the lowest overall discrimination and classification performance. At the selected thresholds, the positive predictive value was low for all models.

**Table 2.** Initial model performance in 2013 test set among the full population.

Metric	Mortality	Readmission	Pneumonia
AUC	0.877 [0.858-0.895]	0.760 [0.751-0.768]	0.851 [0.838-0.864]
Brier	0.009 [0.008-0.01]	0.074 [0.072-0.077]	0.019 [0.018-0.020]
OE	0.920 [0.837-1.010]	0.976 [0.943-1.009]	0.923 [0.864-0.986]
ICI	0.003 [0.002-0.004]	0.009 [0.007-0.011]	0.006 [0.005-0.007]
Sensitivity	0.818 [0.773-0.855]	0.694 [0.678-0.710]	0.722 [0.692-0.754]
Specificity	0.775 [0.771-0.780]	0.678 [0.673-0.684]	0.810 [0.806-0.814]
PPV	0.037 [0.033-0.041]	0.181 [0.173-0.188]	0.082 [0.076-0.089]
NPV	0.998 [0.997-0.998]	0.956 [0.953-0.959]	0.992 [0.991-0.993]
Accuracy	0.776 [0.772-0.780]	0.680 [0.675-0.685]	0.808 [0.804-0.812]

Initial fairness gaps by model are displayed in Table 3, and details of initial performance in each subpopulation are included in Table S1. Performance estimates for female patients had wide confidence intervals due to small sample sizes. Brier scores were lower for female vs male patients in all models. Specificity and accuracy were higher for female vs male patients in all models and for Black vs White patients in models for mortality and pneumonia. Sensitivity was lower for female vs male patients in models for readmission and pneumonia. We did not observe differences in AUC, O:E, or ICI by self-reported race or sex.

**Table 3.** Initial algorithmic fairness as measured by the gap (ie, difference) in metric values across subpopulations.

	Race-based gaps (Black-White)			Sex-based gaps (female-male)		
Metric	Mortality	Readmission	Pneumonia	Mortality	Readmission	Pneumonia
AUC	0.048 [−0.005 to 0.094]	0.011 [−0.012 to 0.032]	0.016 [−0.022 to 0.055]	0.023 [−0.218 to 0.122]	−0.019 [−0.063 to 0.017]	−0.047 [−0.141 to 0.035]
Brier	−0.001 [−0.004 to 0.001]	0.001 [−0.004 to 0.007]	−0.003 [−0.007 to 0]	−0.006 [−0.008 to −0.004]	−0.019 [−0.027 to −0.011]	−0.008 [−0.012 to −0.005]
OE	−0.078 [−0.300 to 0.147]	−0.003 [−0.078 to 0.079]	−0.111 [−0.259 to 0.037]	−0.341 [−0.674 to 0.087]	−0.077 [−0.204 to 0.051]	−0.014 [−0.277 to 0.258]
ICI	0.002 [0 to 0.004]	0.003 [−0.003 to 0.009]	0.003 [0 to 0.006]	0 [−0.001 to 0.002]	0.004 [−0.004 to 0.012]	0.004 [0 to 0.008]
Accuracy	0.020 [0.007 to 0.031]	−0.005 [−0.019 to 0.008]	0.013 [0.003 to 0.023]	0.125 [0.112 to 0.137]	0.096 [0.08 to 0.113]	0.09 [0.078 to 0.102]
Sensitivity	0.054 [−0.051 to 0.156]	0.020 [−0.024 to 0.061]	0.034 [−0.052 to 0.114]	0.086 [−0.225 to 0.215]	−0.138 [−0.222 to −0.073]	−0.179 [−0.344 to −0.014]
Specificity	0.020 [0.007 to 0.031]	−0.008 [−0.022 to 0.006]	0.012 [0.002 to 0.023]	0.125 [0.112 to 0.138]	0.114 [0.097 to 0.131]	0.093 [0.081 to 0.105]
NPV	0.001 [−0.001 to 0.002]	0 [−0.007 to 0.008]	0.002 [−0.001 to 0.004]	0.002 [0.001 to 0.003]	0.006 [−0.003 to 0.015]	0.001 [−0.002 to 0.005]
PPV	0.003 [−0.008 to 0.015]	0.008 [−0.010 to 0.027]	0 [−0.016 to 0.018]	−0.010 [−0.026 to 0.011]	−0.025 [−0.054 to 0.002]	−0.015 [−0.040 to 0.018]

Ideal gap value for all metrics is 0. Gaps that deviated from the ideal value in either direction are highlighted by gray cells.

**Figure 2.**
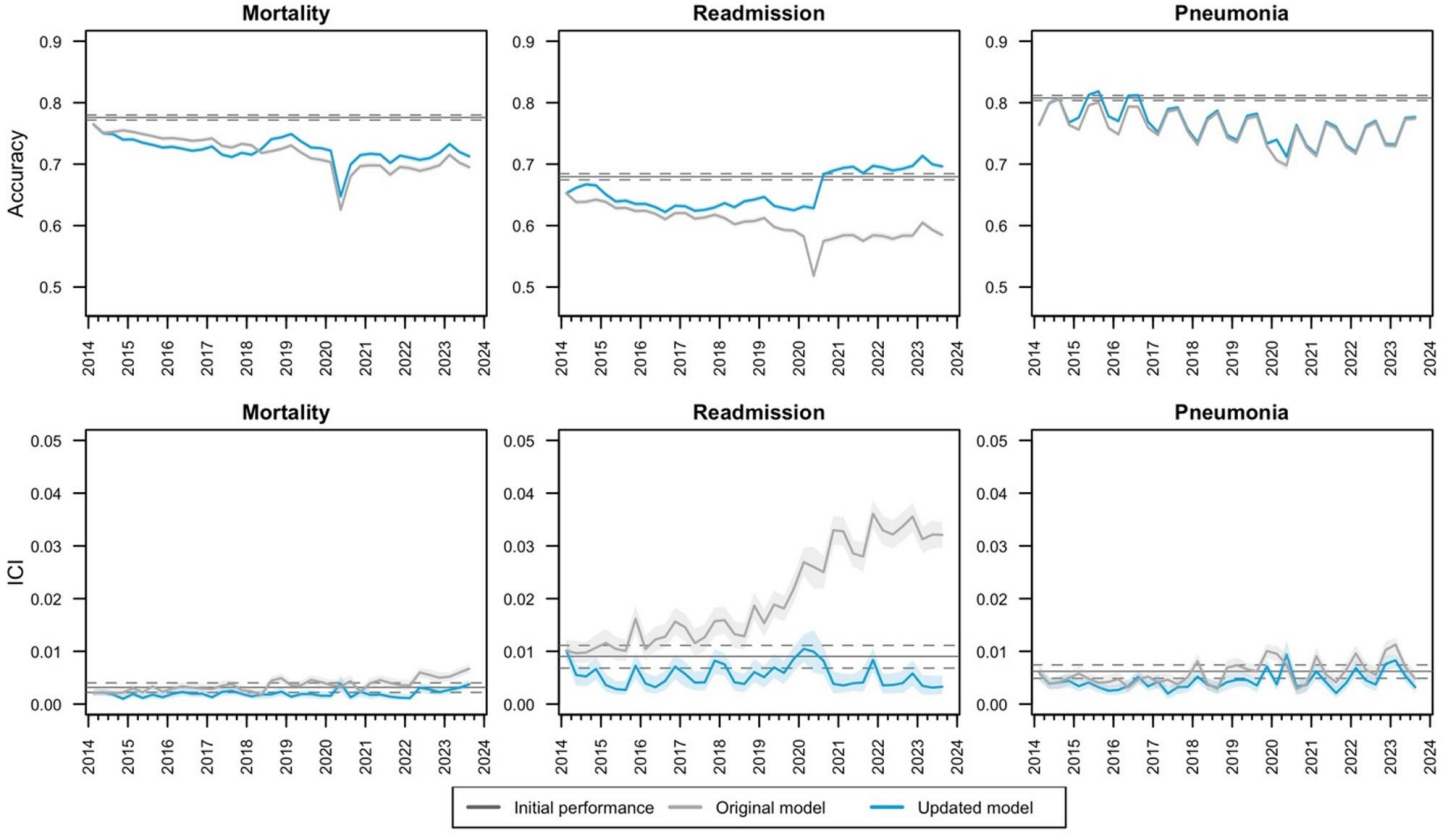
Accuracy (ideal value = 1; top row) and integrated calibration index (ideal value = 0; bottom row) over time in full population with and without surveillance-based updating of random forest models for mortality, readmission, and pneumonia within 30 days of a surgical procedure.

Over the 10-year temporal evaluation period from 2014 to 2023, each of the 3 models experienced temporal drift across multiple metrics. The surveillance-based model update process targeting stabilization of population-level calibration led to the mortality model being recalibrated twice (2014 and 2018), the readmission model being recalibrated 6 times (twice in both 2014 and 2020, as well as once in 2017 and 2018), and the pneumonia model being recalibrated 4 times (2014, 2016, and twice in 2020). Details of the applied model updates are provided in Figure S2. Figure 2 presents the overall performance drift and the impact of the model updating process on accuracy and calibration (see Figure S3 for additional metrics). Seasonal variation in accuracy of the pneumonia model remained even with month of surgery included as a candidate predictor; however, seasonal patterns in calibration were less significant. Accuracy and calibration deteriorated over time in all models, with the largest population-level performance changes observed for the readmission model. The surveillance-based model updating process stabilized calibration of the mortality and pneumonia models but did not restore accuracy. For the readmission model, the surveillance-based model updating process restored both accuracy and calibration to levels comparable or better than initial performance. Sensitivity of the model predicting readmission, which improved over time for the original model, was reduced as a result of model updating to levels below that of initial performance (see Figure S3).

**Figure 3.**
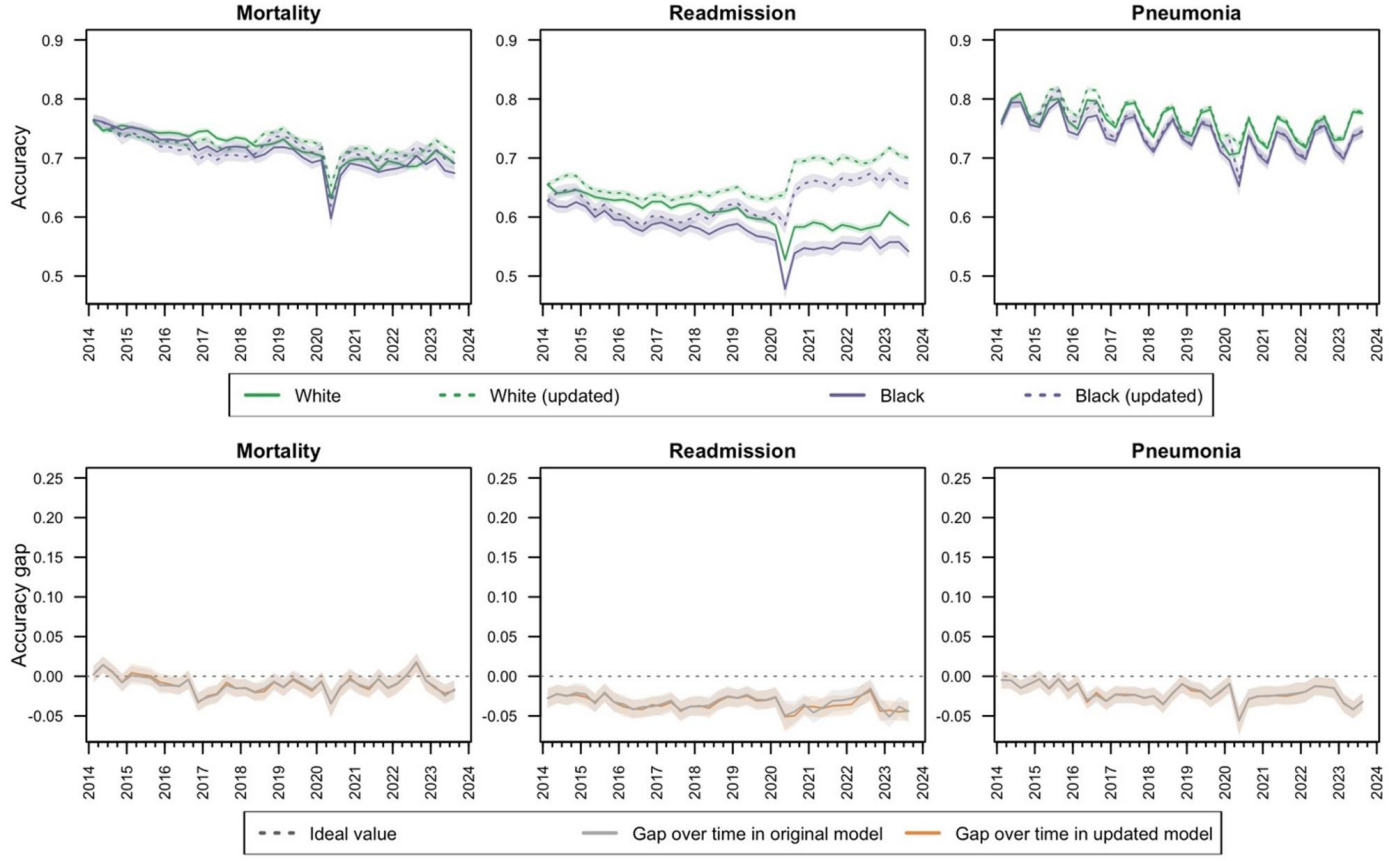
Accuracy (ideal value = 1; top row) and accuracy gap (ideal value = 0; bottom row) by self-reported race with and without population-level surveillance-based updating of random forest models for mortality, readmission, and pneumonia within 30 days of a surgical procedure.

**Figure 4.**
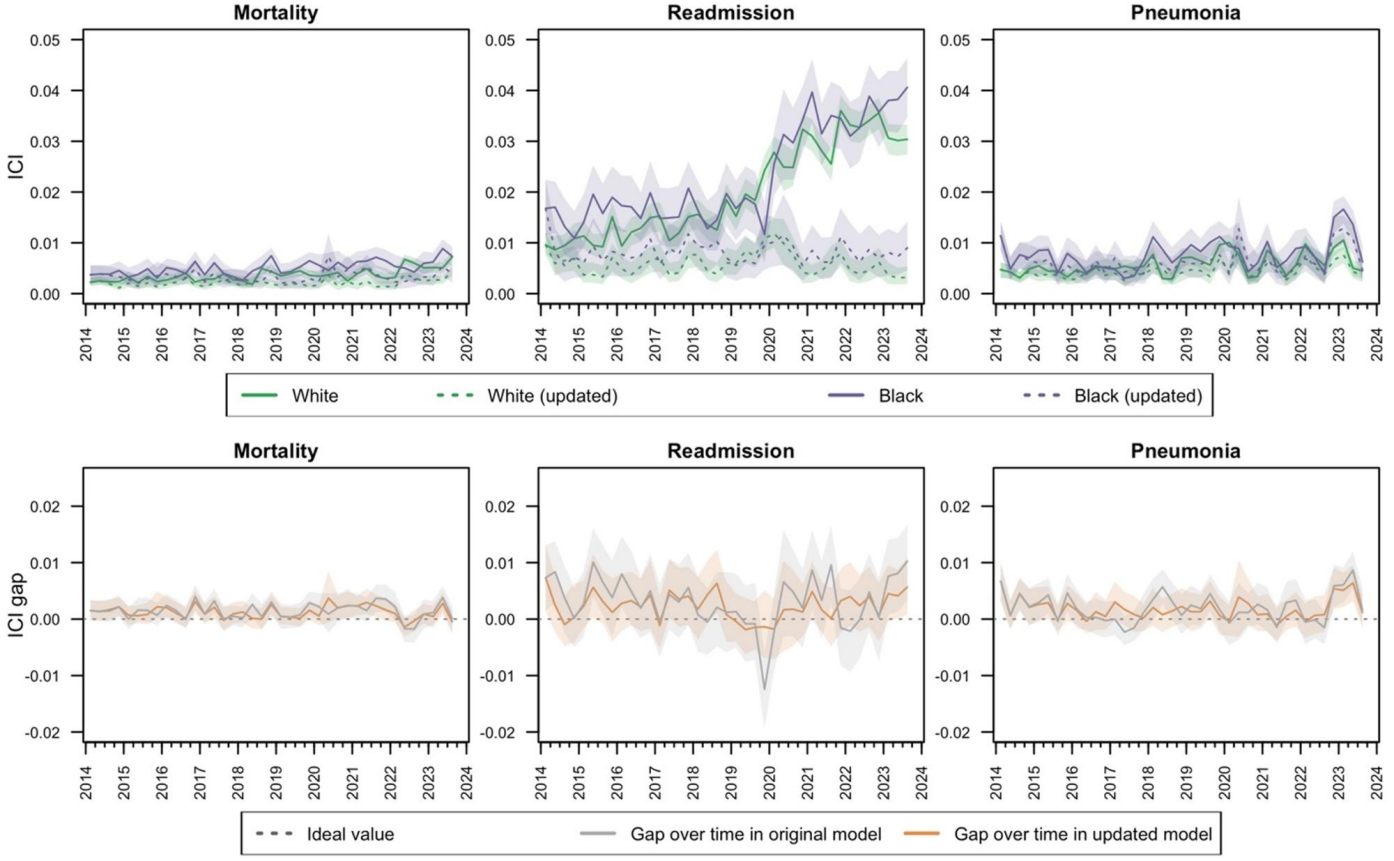
Integrated calibration index (ideal value = 0; top row) and integrated calibration index gap (ideal value = 0; bottom row) by self-reported race with and without population-level surveillance-based updating of random forest models for mortality, readmission, and pneumonia within 30 days of a surgical procedure.

There was evidence of fairness drift by self-reported race when modeling some outcomes but not others (Figures 3 and 4; Figure S4). For the pneumonia model, temporal fairness by self-reported race deteriorated in terms of accuracy and specificity (*P* < .01 for trend in gap in both metrics). Over time, accuracy and specificity declined for both Black and White patients, but the trajectory of this decline was larger for Black patients, leading to an increasing magnitude of the fairness gap for these metrics (*P* < .01 for trend in gap in both metrics). The model updating process improved performance in both subpopulations but did not close the fairness gap that had emerged (*P* < .01 for trend in gap in both metrics). The model updating process corrected calibration drift in both populations in all models and reduced the variability of the ICI gap for the readmission model. With the exception of an increase in the ICI and O:E gap during 2023 for the pneumonia and readmission models, calibration fairness by self-reported race was generally stable over time with and without model updating (*P*-value range = .11-.97 for trends in gap for all calibration metrics).

**Figure 5.**
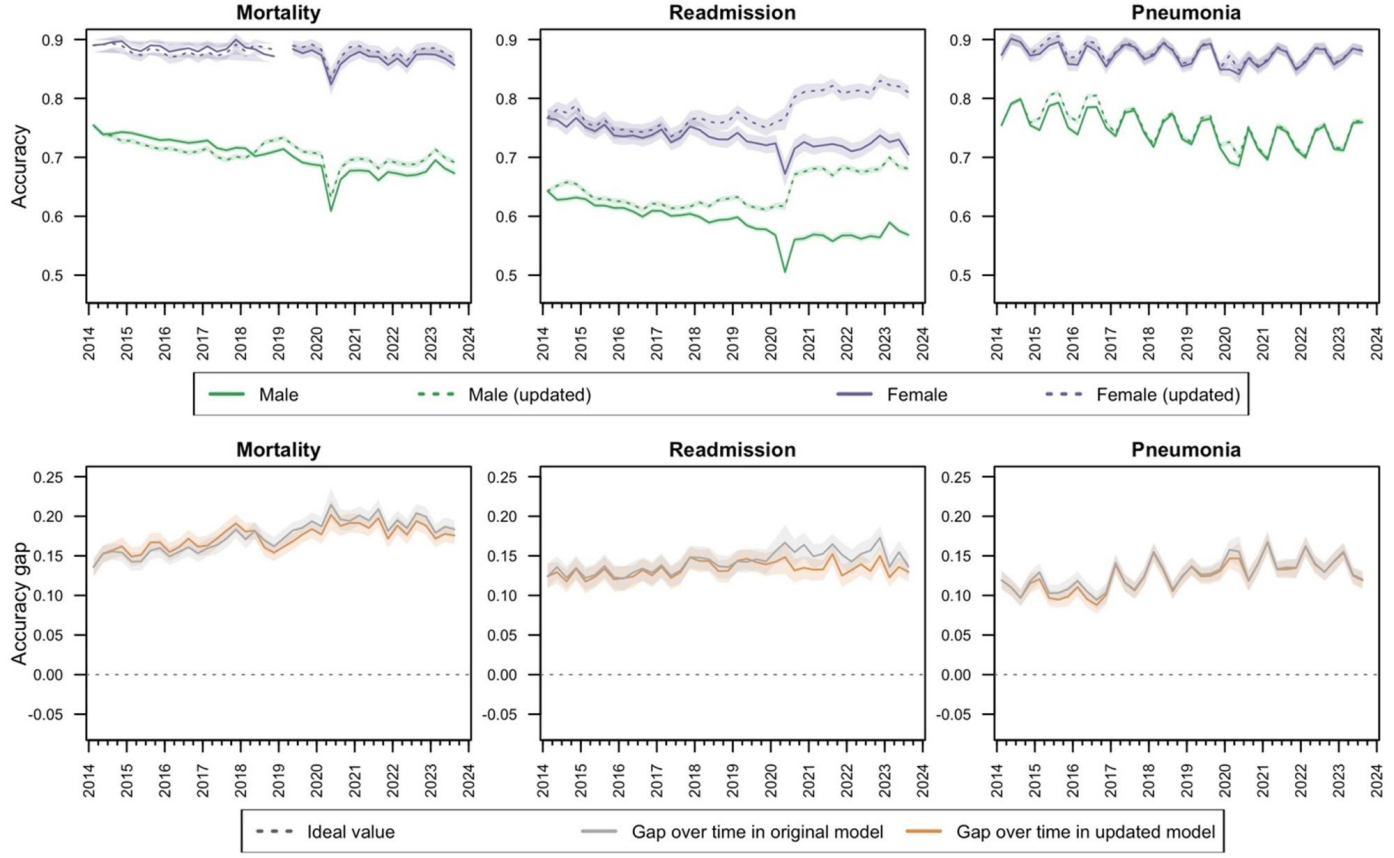
Accuracy (ideal value = 1; top row) and accuracy gap (ideal value = 0; bottom row) by self-reported sex with and without population-level surveillance-based updating of random forest models for mortality, readmission, and pneumonia within 30 days of a surgical procedure.

**Figure 6.**
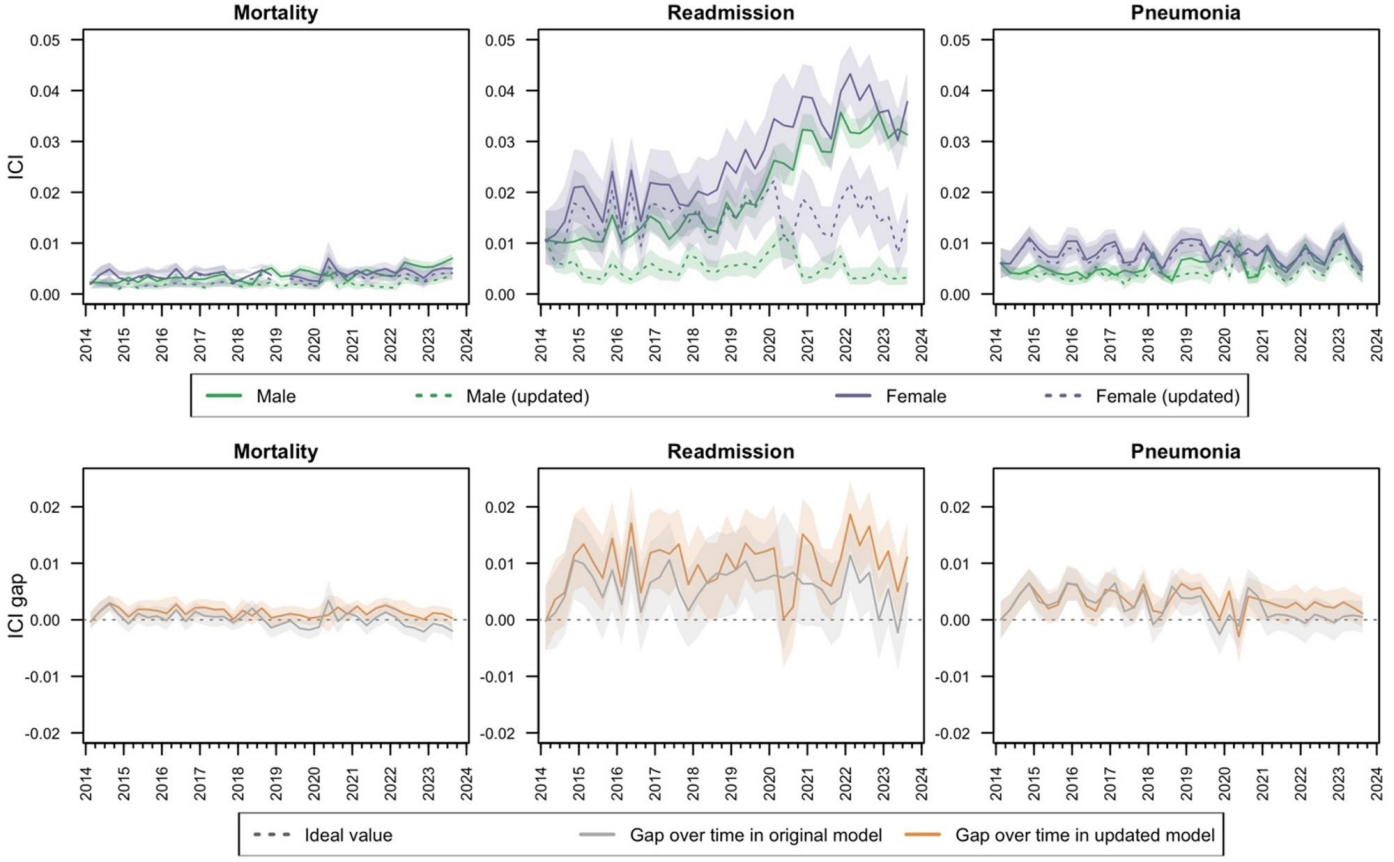
Integrated calibration index (ideal value = 0; top row) and integrated calibration index gap (ideal value = 0; bottom row) by self-reported sex with and without population-level surveillance-based updating of random forest models for mortality, readmission, and pneumonia within 30 days of a surgical procedure.

We observed fairness drift by self-reported sex (Figures 5 and 6; Figure S5). Accuracy and specificity drifted in all models for both female and male patients (*P* < .01 for trends in both metrics). The surveillance-based model updating process restored or improved these metrics in both subpopulations. Over time, the accuracy and specificity gaps between female and male patients increased in the original models for all 3 outcomes (*P* < .01 for trends in gap in both metrics). Based on visual inspection and trend assessments, surveillance-based model updating reduced these gaps to near their initial magnitude for the readmission model (*P* < .01 for interaction in gap trend between time and model maintenance; *P* = .01 for trend in accuracy gap and *P* = .56 for trend in specificity gap with model updating), reduced the increase in gap magnitude but did not fully restore fairness to initial levels for the mortality model (*P* = .01 for interaction in gap trend between time and model maintenance; *P* < .1 for trend in gap in both metrics with model updating), and did not affect these gaps for the pneumonia model (*P* = .73-.75 for interaction in gap trend between time and model maintenance). In the original models, performance drift resulted in increased calibration fairness as ICI values become more similar for female and male patients over time (*P* < .01 for trend in gap for original pneumonia and mortality models). In the model for readmission, the model updating process corrected ICI drift for both subpopulations, but the improvement in calibration was larger for males, leading to an ICI gap that trended larger (though not statistically significant) compared to the original model in later years. In the model for pneumonia, ICI of the original model was stable in the female population (*P* = .76) and deteriorating in the male population (*P* < .01), resulting in a narrowing over time of the gap in ICI for the original model (*P* < .01). Surveillance-based model updating improved ICI more for the male population than the female population. This resulted in stable calibration fairness over time under the surveillance-based model updating process (*P* = .14) despite this approach exhibiting a larger ICI gap compared to the original model in later years.

## Discussion

This study begins to illuminate the challenge of maintaining algorithmic fairness over time in evolving clinical environments and highlights the need for and difficulty of integrating fairness sustainability into comprehensive algorithmic vigilance programs.^31^ Using an example based on the ACS NSQIP universal risk calculators, tools designed to support patient and provider decision-making around the potential risk of surgical procedures, we observed multiple, complex patterns of both population-level performance drift and fairness drift over 10 years. We found that population-level performance drift may result from similar drift patterns across subpopulations, leading to overall deterioration in performance alongside stable algorithmic fairness. This pattern was noted in the model for post-surgical readmission, where race-based fairness metrics remained stable despite population-level drift. We also found that population-level performance drift may result from changes predominantly affecting performance in one subpopulation, leading to fairness drift that results in the widening or narrowing of fairness gaps. This pattern was observed in sex-based fairness evaluations of the post-surgical pneumonia model in which calibration deteriorated for males more so than for females, leading to more similar performance between the 2 groups and improved fairness over time.

Complicating the situation, however, our understanding of fairness drift is closely tied to the fairness metrics considered. For example, while the calibration gap between female and male patients diminished over time in the post-surgical pneumonia model, the accuracy gap actually broadened over time. While contradictory messaging from different fairness metrics is not unexpected and, in some cases, inherent,^2^ this finding underscores the importance of tailoring post-deployment model monitoring around use-case fairness priorities.

As model maintenance strategies to address performance drift have thus far focused on restoring and stabilizing population-level model performance, we further explored the impact of population-level data-driven surveillance-based model updating on algorithmic fairness. While managing the model updating process at the population level had a more substantial impact on population-level performance than on algorithmic fairness, we did observe interactions between the model updating process and fairness metrics that complicate future efforts to sustain fair AI tools. In some cases, such as the post-surgical readmission model, the surveillance-based model updating process aiming to stabilize population-level performance did not impact the magnitude of the fairness gap in accuracy or calibration between Black and White patients. However, updates applied to the post-surgical pneumonia model disproportionately improved performance for the male subpopulation, restoring the fairness gap that had been diminishing due to performance drift. This finding is likely related to the dominant size of the male subpopulation relative to the female subpopulation (1 580 989 cases involving males and 158 677 cases involving females)—such situations with highly disparate samples sizes may be common across some health equity dimensions and require innovative model monitoring and maintenance approaches that account for subpopulation performance. Further complicating the situation, the gap in accuracy between female and male patients for the original pneumonia model broadened over time, and the model updating process alleviated some of this fairness drift.

While the patterns and trends observed in this study are exploratory, our results highlight the need for further investigation of fairness drift and the development of recommendations for sustaining algorithmic fairness over time. As we observed, existing algorithmic vigilance methods that focus on population-level data—monitoring only overall performance and assessing model updates in the full population—may fail to address fairness drift and may even unintentionally exacerbate algorithmic fairness concerns in some cases. More comprehensive algorithmic vigilance programs will need to explicitly establish fairness expectations for each model and use case, as well as guidelines for managing trade-offs between overall and subpopulation model performance, especially when the benefits of model updating are not evenly realized across the entire population. This will likely require the development of novel fairness-informed drift detection and model updating methods that guide model maintenance recommendations to sustain population-level performance while ensuring subpopulation performance is not compromised over time.

We note several limitations of this study. First, the granular nature of self-reported race and sex information in the structured VA data limited the specificity of subpopulation analyses. Second, even in a large national cohort of veterans receiving care at the VA, sample sizes for some groups diminish quickly when divided over multiple temporal windows. As a result, we were unable to explore race-based fairness among patients not identifying as either Black or White. The experience of and factors influencing health outcomes and model performance for more nuanced patient subpopulations may be important for improving health equity and preventing clinical AI from exacerbating inequities. However, being restricted to the more granular subpopulation pairs of self-reported Black vs White race and female vs male sex is sufficient for the exploratory nature of this study aiming to develop an understanding of the variations and challenges of temporal fairness drift. Finally, this exploration of algorithmic fairness drift focuses on model performance. We note that fairness drift may also manifest in temporal changes to input data biases or temporal shifts in how implementation decisions may influence the fairness of model impacts on patient outcomes over time. This complexity further highlights the need for more research to comprehensively sustain fair algorithms.

## Conclusion

In this study, we highlight 2 challenges to sustainable, equitable AI deployments in healthcare. First, that algorithmic fairness, like other dimensions of model performance, cannot be assured through one-time assessments at the time of model development and temporal changes in algorithmic fairness may take multiple, complex forms. Second, algorithmic fairness may interact with model updates in unanticipated ways when maintenance strategies are designed at the population level. While this analysis focused on fairness across self-reported race and sex, fairness drift across geographic, social, and clinical subpopulations may also affect the impact of clinical AI tools. Comprehensive post-deployment AI monitoring and maintenance programs should incorporate fairness evaluations across multiple subpopulations based on known health inequities and in consultation with providers and patients. Equitable and sustainable clinical AI deployments will require novel methods to monitor algorithmic fairness, detect emerging bias, and adopt updates that promote and restore fairness.

## Supplementary Material

ocaf039_Supplementary_Data

## Data Availability

The data underlying this article cannot be shared publicly due to privacy and disclosure requirements of the US Departments of Veterans Affairs. All analytic codes can be shared, and instructions for investigators can be accessed. VA data to be able to reproduce the data will be shared on as permissible through reasonable request to the corresponding author.
