# Supplementary material for: Emerging algorithmic bias: fairness drift as the next dimension of model maintenance and sustainability
Source: J Am Med Inform Assoc. 2025 Mar 13;32(5):845–54. doi: 10.1093/jamia/ocaf039 (PMC12012346; doi:10.1093/jamia/ocaf039)

**Appendix Table 1.** Feature list for ACS NSQIP models and models presented in this paper.

| **Predictive features** | **Included in ACS NSQIP** | **Included in our models** | **Notes** |
| --- | --- | --- | --- |
| Primary procedure CPT code | ✓ | ✓ |  |
| Age | ✓ | ✓ | NSQIP categorized, we used continuous values |
| Sex | ✓ | ✓ |  |
| BMI | ✓ | ✓ | NSQIP categorized, we used continuous values |
| Sepsis in prior 48hrs | ✓ |  |  |
| Ventilator dependent | ✓ |  |  |
| Emergency case | ✓ |  |  |
| Ascites | ✓ | ✓ |  |
| Acute renal failure | ✓ | ✓ |  |
| History of COPD | ✓ | ✓ | We defined using Elixhauser definition |
| ASA class | ✓ |  |  |
| Congestive heart failure | ✓ | ✓ | We defined using Elixhauser definition |
| Steroid use for chronic condition | ✓ |  |  |
| Hypertension requiring medication | ✓ | ✓ | We defined using Elixhauser definition |
| Current smoker within 1y | ✓ | ✓ |  |
| Diabetes | ✓ | ✓ | We defined using Elixhauser diabetes-related flags |
| Functional status | ✓ |  |  |
| Disseminated cancer | ✓ | ✓ | We defined using Elixhauser cancer-related flags (lymphoma, metastatic cancer, solid tumor metastasis) |
| Dialysis | ✓ | ✓ |  |
| Previous cardiac event (cardiac arrest or mycardial infarction) | ✓ | ✓ | We defined using Elixhauser cardiac-related flags |
| Dyspnea | ✓ |  |  |
| Alcohol abuse |  | ✓ | Additional Elixhauser conditions included |
| Valvular disease |  | ✓ | Additional Elixhauser conditions included |
| Pulmonary circulation disorders |  | ✓ | Additional Elixhauser conditions included |
| Peripheral vascular disorders |  | ✓ | Additional Elixhauser conditions included |
| Paralysis |  | ✓ | Additional Elixhauser conditions included |
| Other neurological disorders |  | ✓ | Additional Elixhauser conditions included |
| Chronic pulmonary disease |  | ✓ | Additional Elixhauser conditions included |
| Hypothyroidism |  | ✓ | Additional Elixhauser conditions included |
| Renal failure |  | ✓ | Additional Elixhauser conditions included |
| Liver disease |  | ✓ | Additional Elixhauser conditions included |
| Peptic ulcer disease |  | ✓ | Additional Elixhauser conditions included |
| HIV/AIDS |  | ✓ | Additional Elixhauser conditions included |
| Rheumatoid arthritis |  | ✓ | Additional Elixhauser conditions included |
| Coagulopathy |  | ✓ | Additional Elixhauser conditions included |
| Weight loss |  | ✓ | Additional Elixhauser conditions included |
| Fluid and electrolyte disorders |  | ✓ | Additional Elixhauser conditions included |
| Blood loss anemia |  | ✓ | Additional Elixhauser conditions included |
| Drug abuse |  | ✓ | Additional Elixhauser conditions included |
| Psychosis |  | ✓ | Additional Elixhauser conditions included |
| Depression |  | ✓ | Additional Elixhauser conditions included |

**Appendix Figure 1.** Quarterly outcome rates overall and by subpopulation by quarter, 2013-2023.


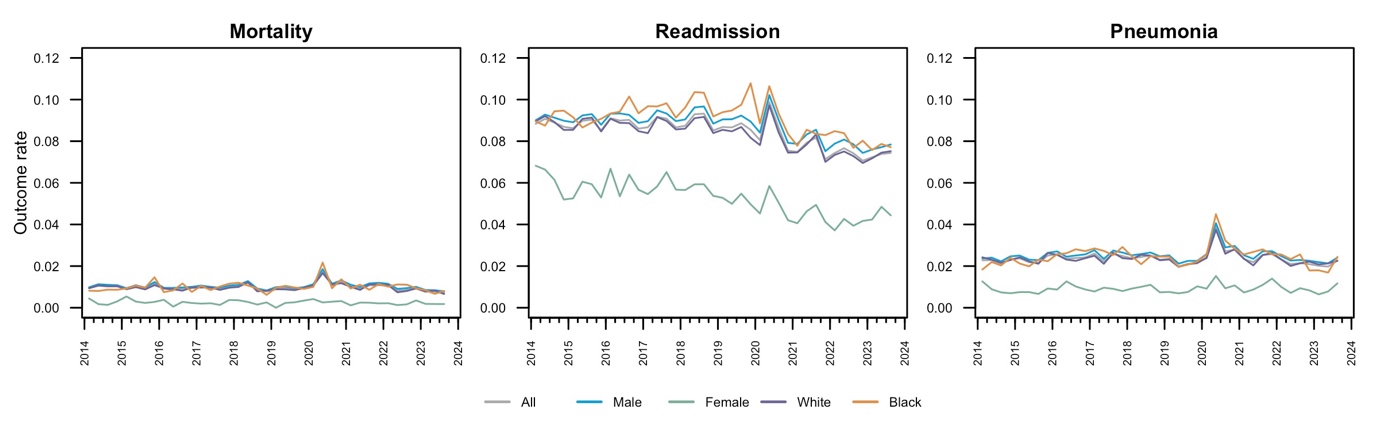


**Appendix Table 2.** Initial model performance on 2013 test set by self-reported race and sex subpopulations.

|  | **Female** | **Male** |  | **Black** | **White** |
| --- | --- | --- | --- | --- | --- |
| *Mortality* |  |  |  |  |  |
| AUC | 0.895 [0.663 - 0.987] | 0.874 [0.853 - 0.893] |  | 0.916 [0.868 - 0.955] | 0.867 [0.845 - 0.888] |
| Brier | 0.003 [0.002 - 0.005] | 0.009 [0.008 - 0.01] |  | 0.007 [0.005 - 0.01] | 0.009 [0.008 - 0.01] |
| OE | 0.592 [0.264 - 1.025] | 0.932 [0.846 - 1.026] |  | 0.837 [0.631 - 1.045] | 0.912 [0.814 - 1.021] |
| ICI | 0.004 [0.003 - 0.005] | 0.003 [0.002 - 0.004] |  | 0.005 [0.003 - 0.007] | 0.003 [0.002 - 0.004] |
| Sensitivity | 0.9 [0.6 - 1] | 0.816 [0.77 - 0.855] |  | 0.86 [0.763 - 0.949] | 0.804 [0.752 - 0.847] |
| Specificity | 0.891 [0.879 - 0.902] | 0.766 [0.761 - 0.77] |  | 0.791 [0.781 - 0.802] | 0.772 [0.767 - 0.777] |
| PPV | 0.027 [0.01 - 0.048] | 0.037 [0.033 - 0.041] |  | 0.038 [0.028 - 0.049] | 0.035 [0.031 - 0.04] |
| NPV | 1 [0.999 - 1] | 0.997 [0.997 - 0.998] |  | 0.998 [0.997 - 0.999] | 0.997 [0.997 - 0.998] |
| Accuracy | 0.891 [0.879 - 0.902] | 0.766 [0.762 - 0.771] |  | 0.792 [0.781 - 0.802] | 0.772 [0.767 - 0.777] |
| *Readmission* |  |  |  |  |  |
| AUC | 0.74 [0.697 - 0.777] | 0.76 [0.751 - 0.769] |  | 0.768 [0.747 - 0.788] | 0.756 [0.746 - 0.767] |
| Brier | 0.057 [0.05 - 0.064] | 0.076 [0.074 - 0.078] |  | 0.076 [0.071 - 0.081] | 0.074 [0.072 - 0.077] |
| OE | 0.902 [0.782 - 1.03] | 0.981 [0.948 - 1.014] |  | 0.975 [0.909 - 1.047] | 0.977 [0.939 - 1.014] |
| ICI | 0.013 [0.005 - 0.021] | 0.009 [0.007 - 0.011] |  | 0.012 [0.006 - 0.017] | 0.009 [0.006 - 0.011] |
| Sensitivity | 0.563 [0.482 - 0.627] | 0.701 [0.685 - 0.718] |  | 0.706 [0.668 - 0.742] | 0.687 [0.669 - 0.707] |
| Specificity | 0.783 [0.767 - 0.799] | 0.669 [0.664 - 0.675] |  | 0.671 [0.658 - 0.683] | 0.679 [0.673 - 0.685] |
| PPV | 0.156 [0.129 - 0.183] | 0.182 [0.175 - 0.19] |  | 0.186 [0.171 - 0.203] | 0.179 [0.171 - 0.187] |
| NPV | 0.962 [0.952 - 0.97] | 0.955 [0.952 - 0.958] |  | 0.955 [0.949 - 0.962] | 0.955 [0.952 - 0.959] |
| Accuracy | 0.769 [0.752 - 0.784] | 0.672 [0.667 - 0.677] |  | 0.674 [0.662 - 0.686] | 0.679 [0.674 - 0.686] |
| *Pneumonia* |  |  |  |  |  |
| AUC | 0.806 [0.717 - 0.887] | 0.852 [0.839 - 0.865] |  | 0.864 [0.827 - 0.898] | 0.848 [0.833 - 0.863] |
| Brier | 0.011 [0.008 - 0.015] | 0.02 [0.018 - 0.021] |  | 0.016 [0.013 - 0.019] | 0.019 [0.018 - 0.021] |
| OE | 0.915 [0.656 - 1.181] | 0.924 [0.862 - 0.987] |  | 0.822 [0.698 - 0.954] | 0.935 [0.869 - 1.004] |
| ICI | 0.01 [0.006 - 0.013] | 0.006 [0.005 - 0.007] |  | 0.009 [0.006 - 0.012] | 0.006 [0.004 - 0.007] |
| Sensitivity | 0.548 [0.385 - 0.714] | 0.73 [0.7 - 0.761] |  | 0.75 [0.672 - 0.825] | 0.716 [0.681 - 0.752] |
| Specificity | 0.896 [0.884 - 0.907] | 0.803 [0.798 - 0.807] |  | 0.819 [0.809 - 0.828] | 0.806 [0.802 - 0.811] |
| PPV | 0.068 [0.043 - 0.099] | 0.083 [0.076 - 0.089] |  | 0.081 [0.066 - 0.097] | 0.081 [0.073 - 0.088] |
| NPV | 0.993 [0.99 - 0.996] | 0.992 [0.991 - 0.993] |  | 0.994 [0.991 - 0.996] | 0.992 [0.99 - 0.993] |
| Accuracy | 0.891 [0.879 - 0.903] | 0.801 [0.797 - 0.805] |  | 0.817 [0.808 - 0.827] | 0.804 [0.8 - 0.809] |

**Appendix Figure 2.** Temporal model updating summary.

**
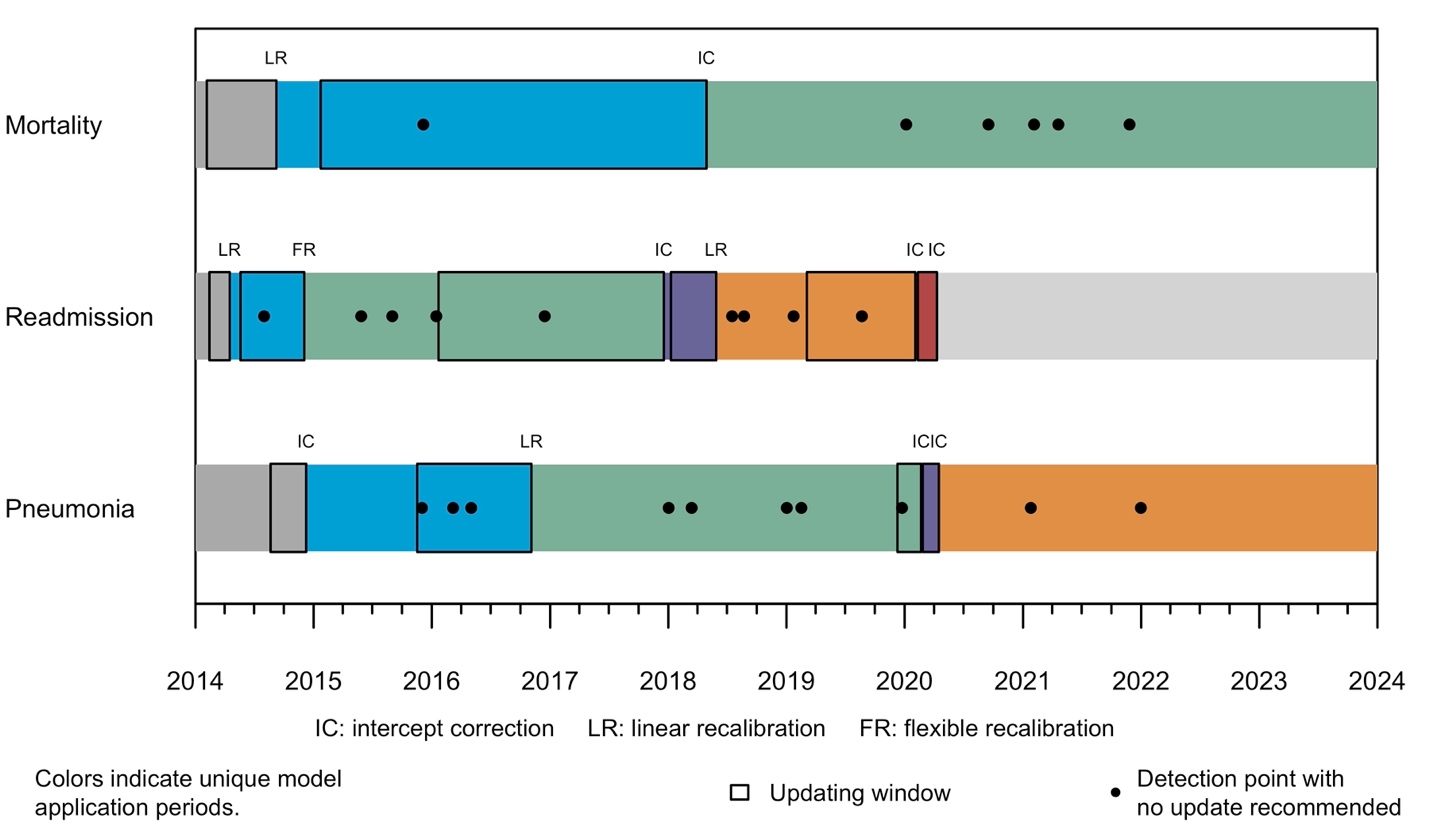
**

**Appendix Figure 3.** Performance metrics over time in full population with and without updating.


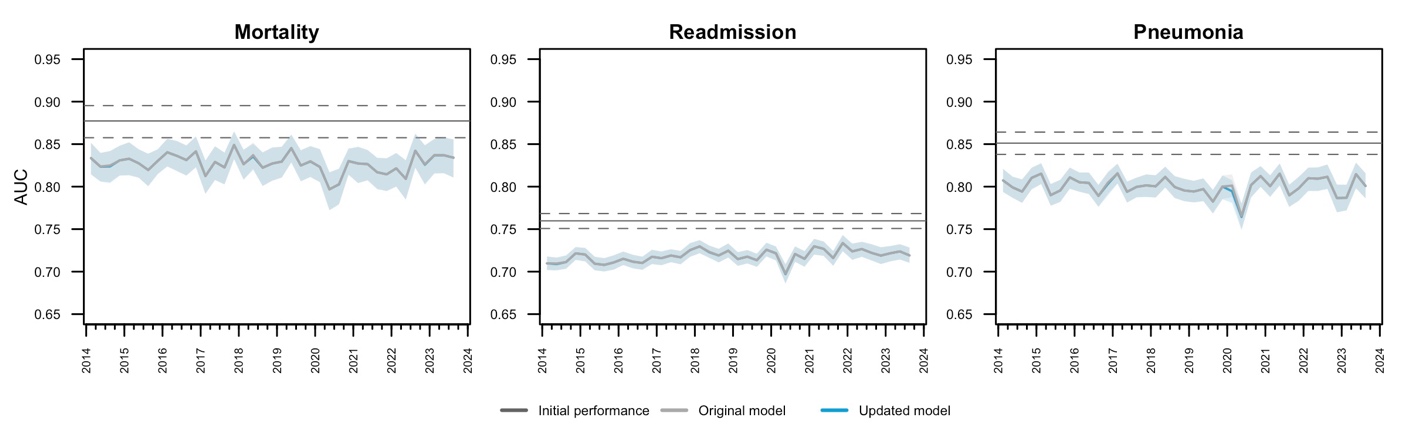


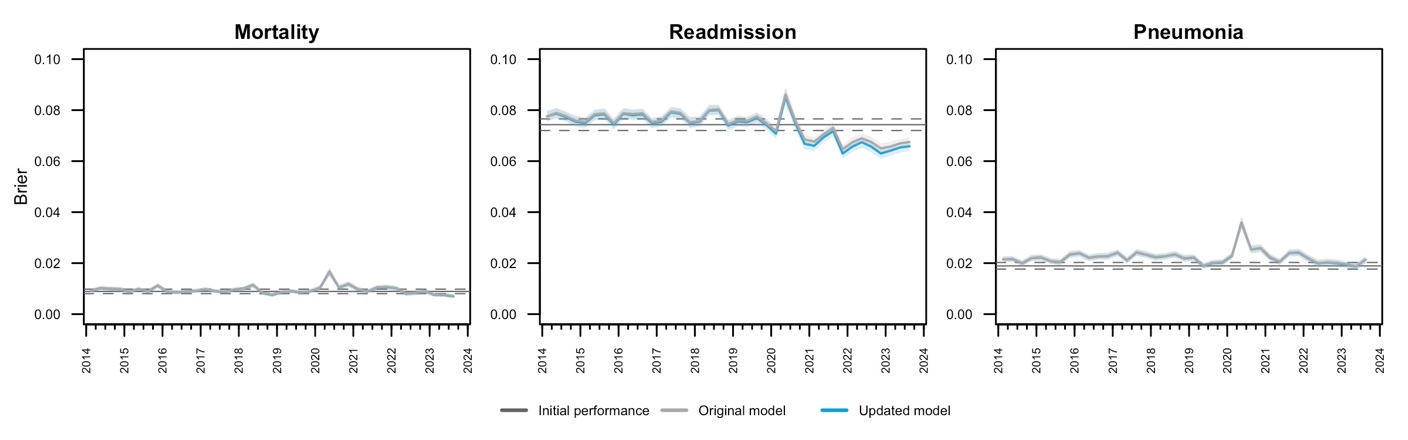


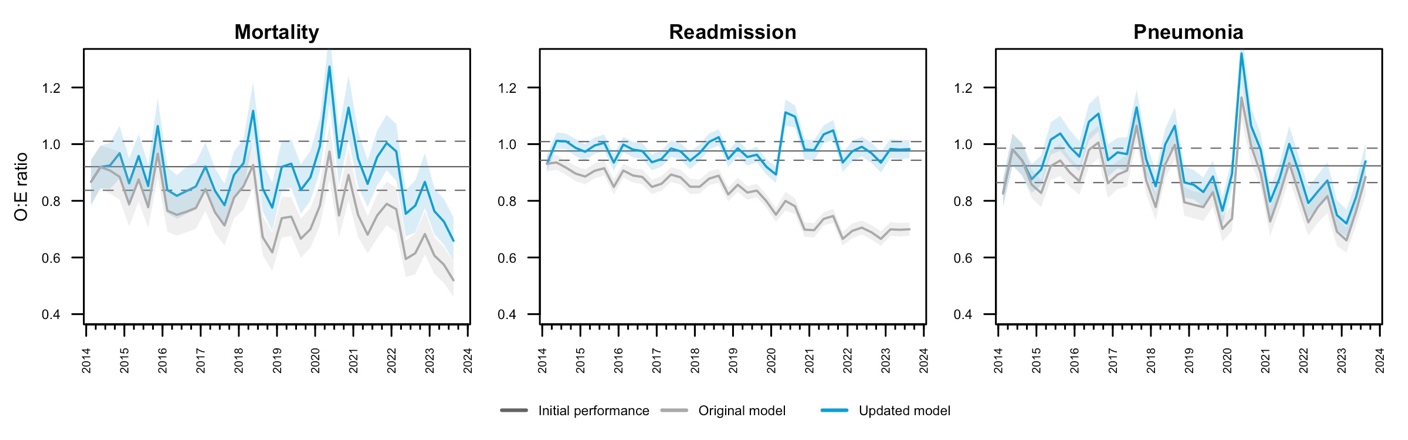


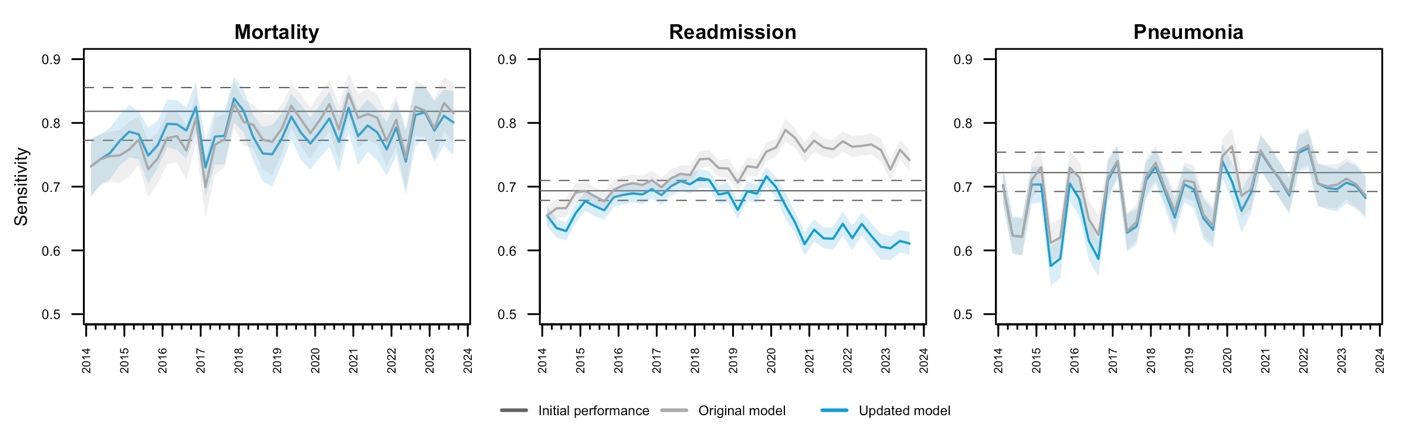


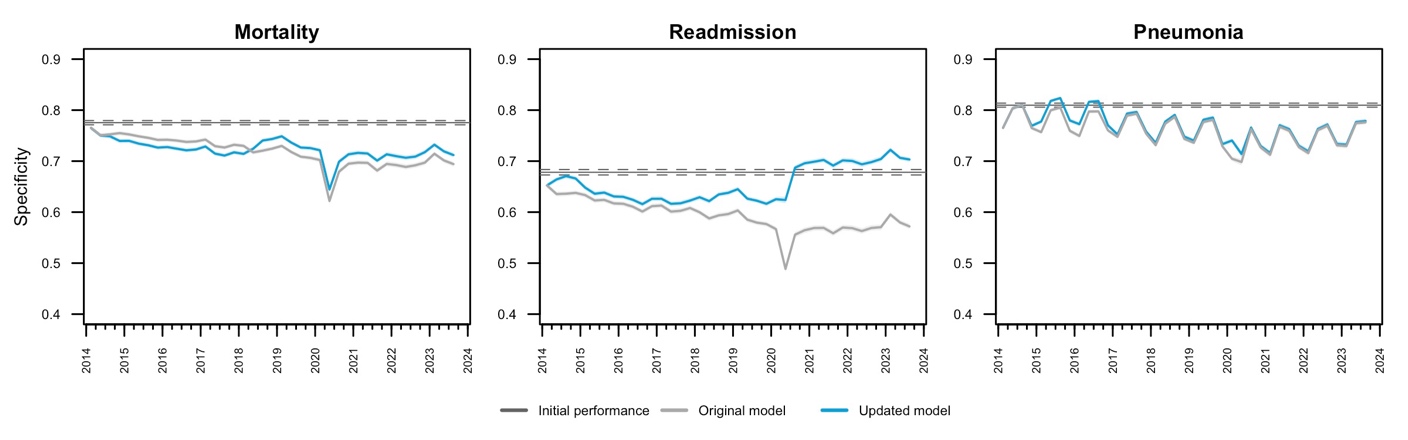


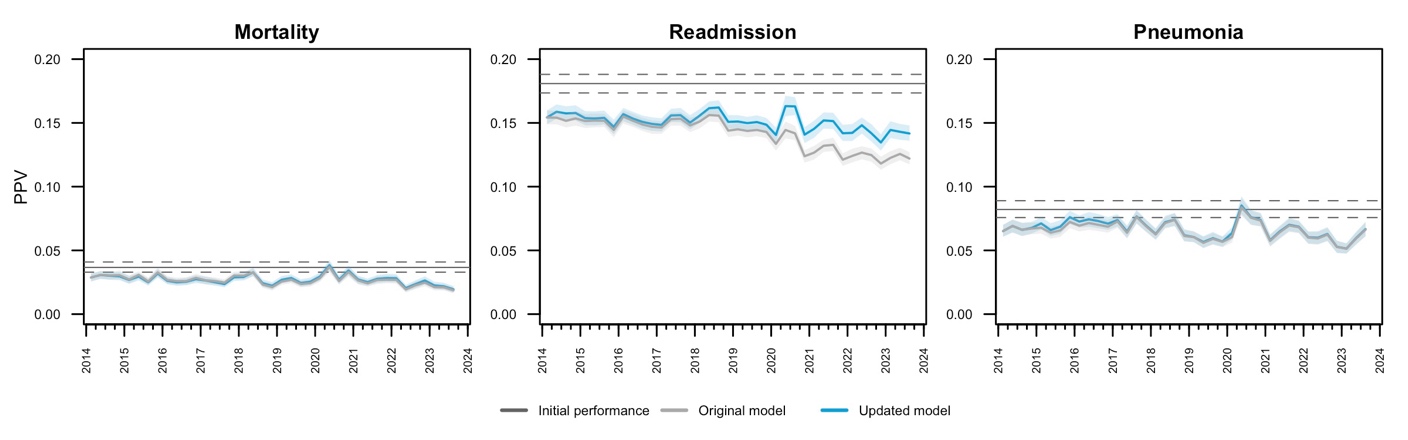


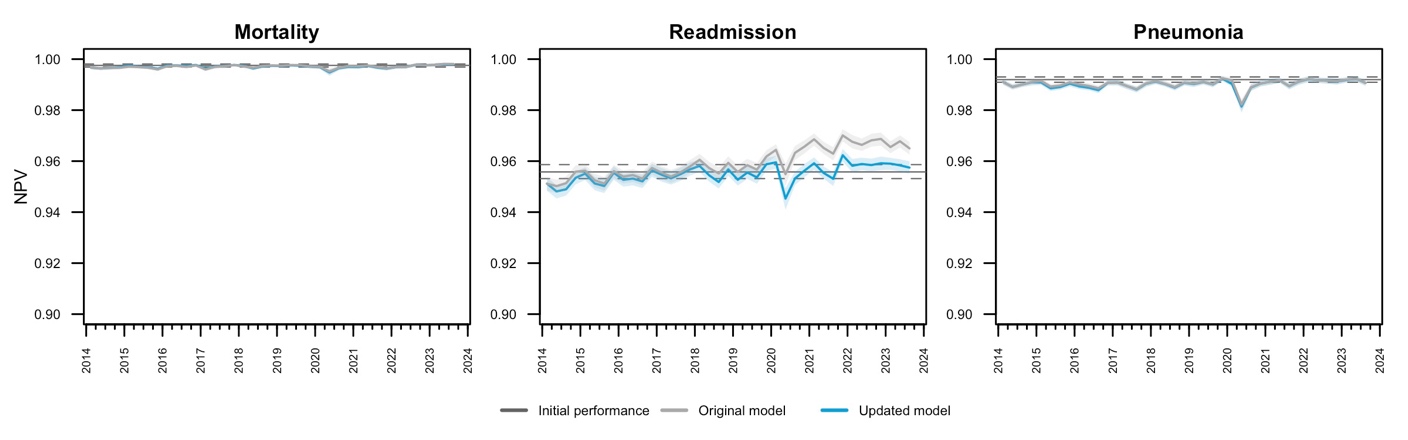


**Appendix Figure 4.** Performance gaps over time by self-reported race with and without model updating.


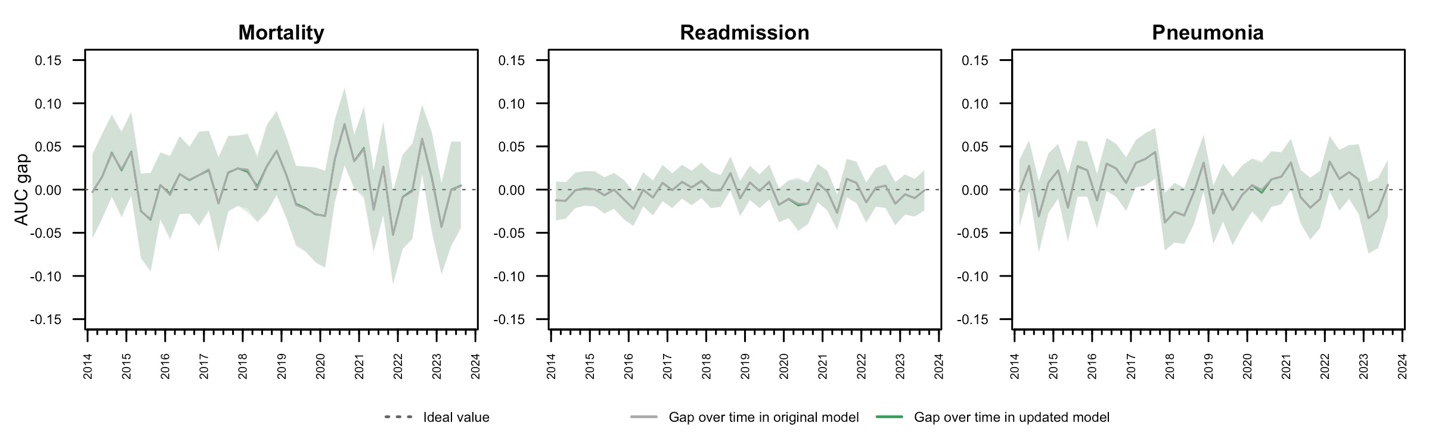

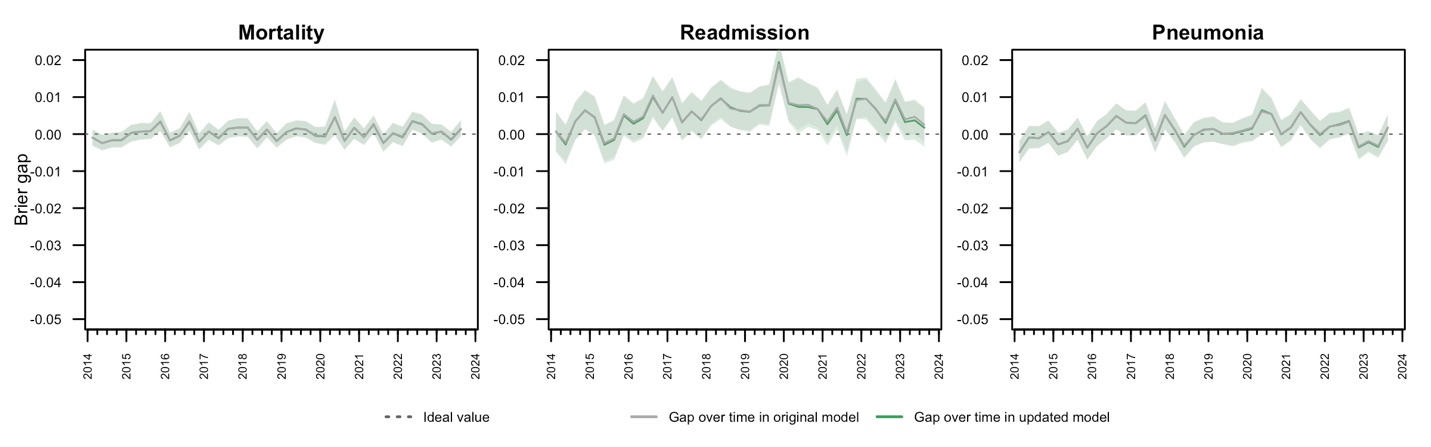

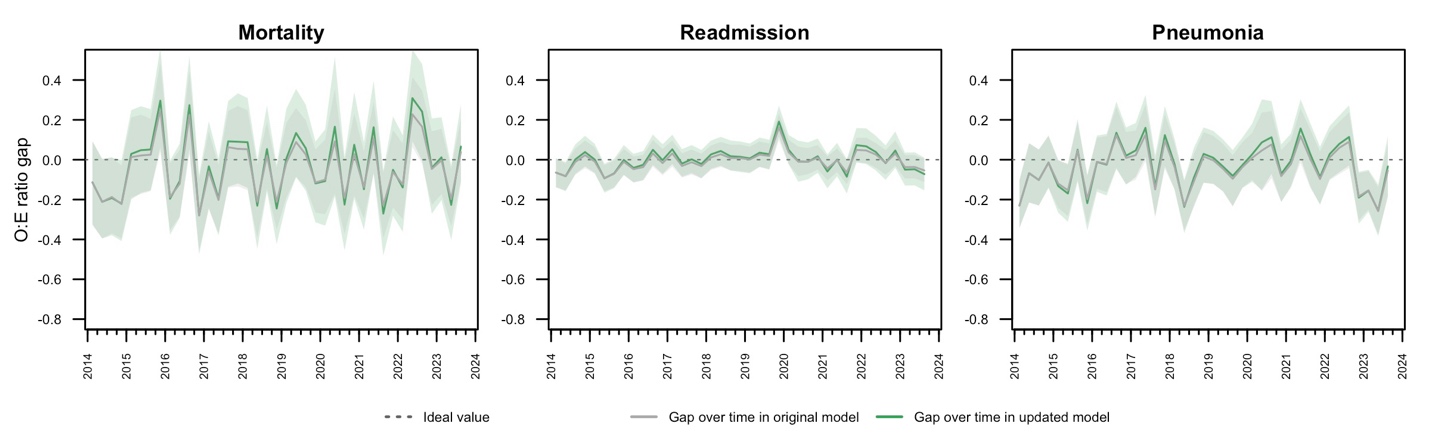

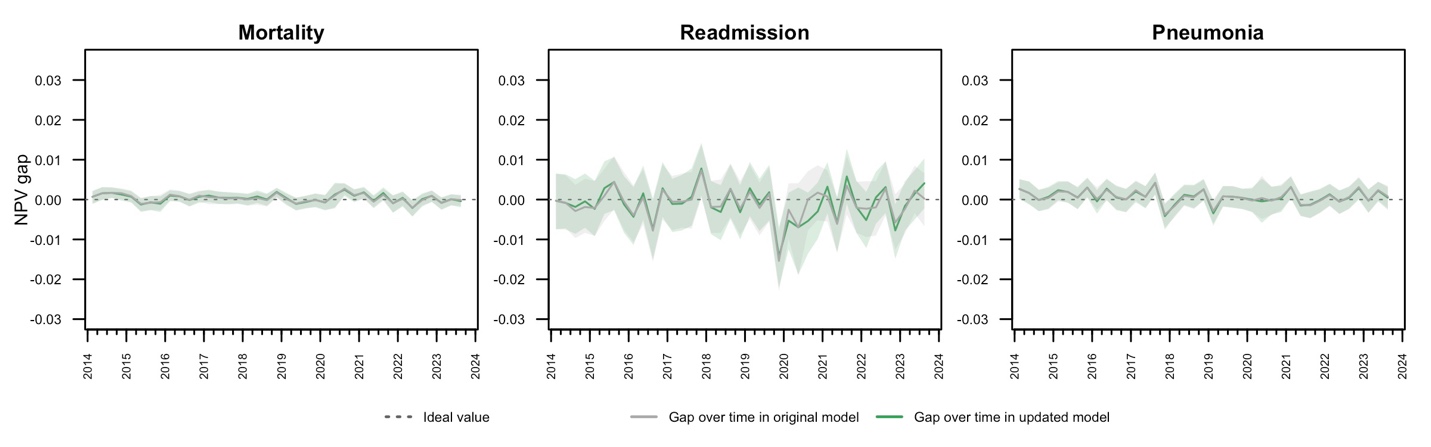

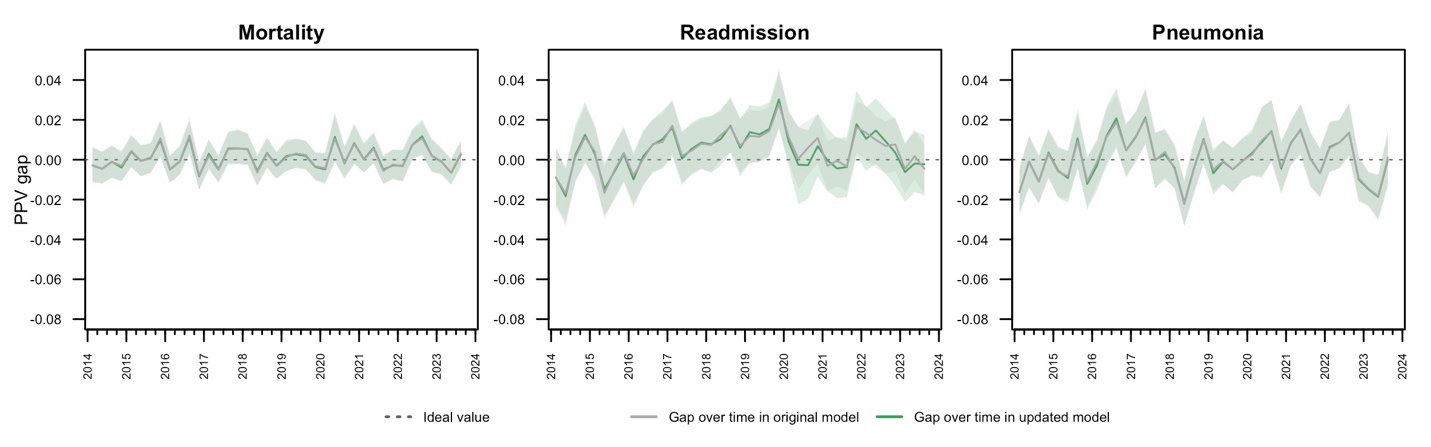

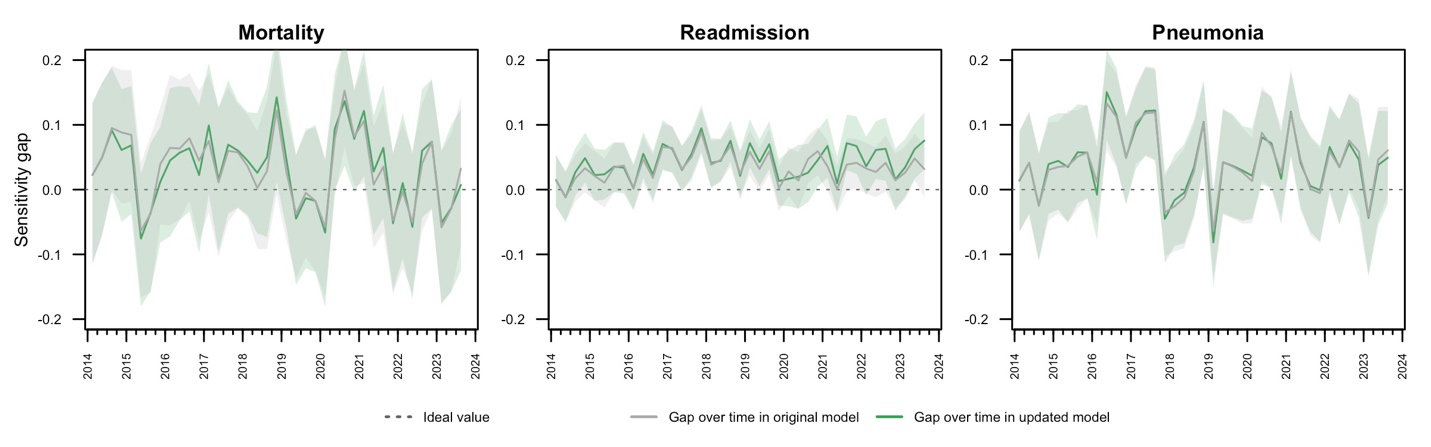

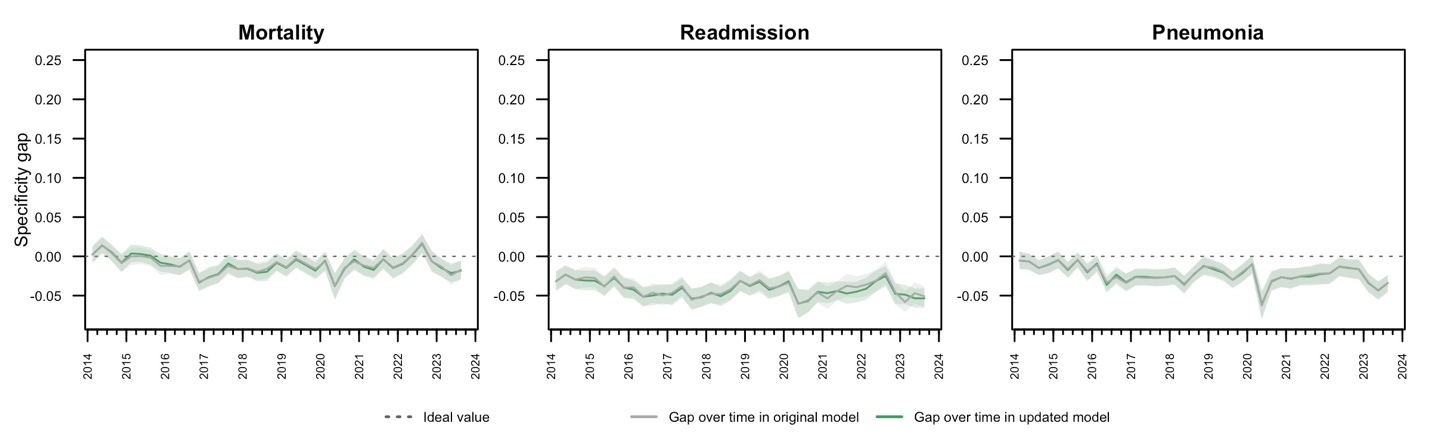


**Appendix Figure 5.** Performance gaps over time by self-reported sex with and without model updating.


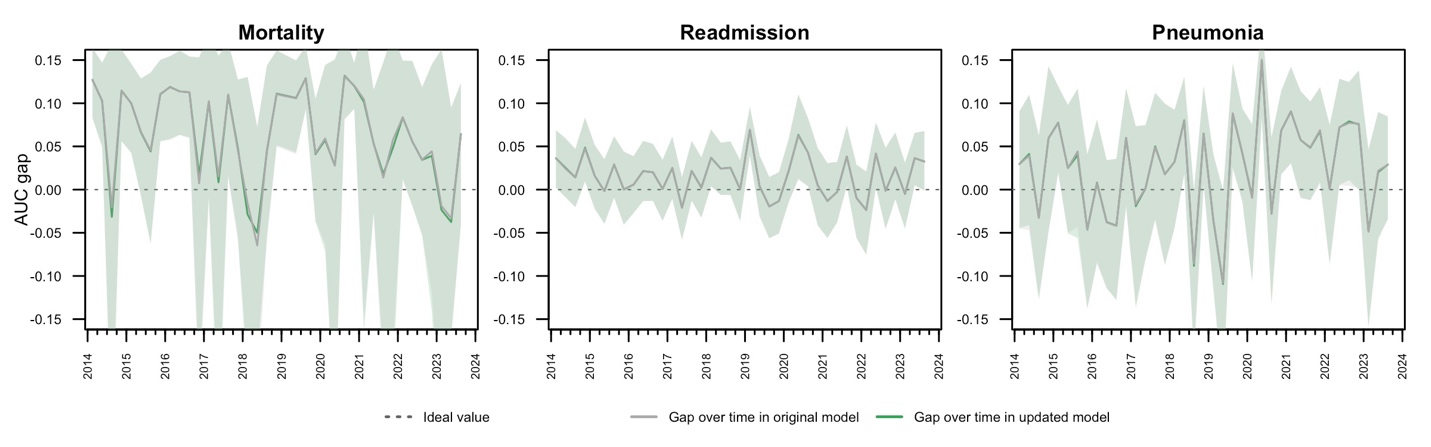

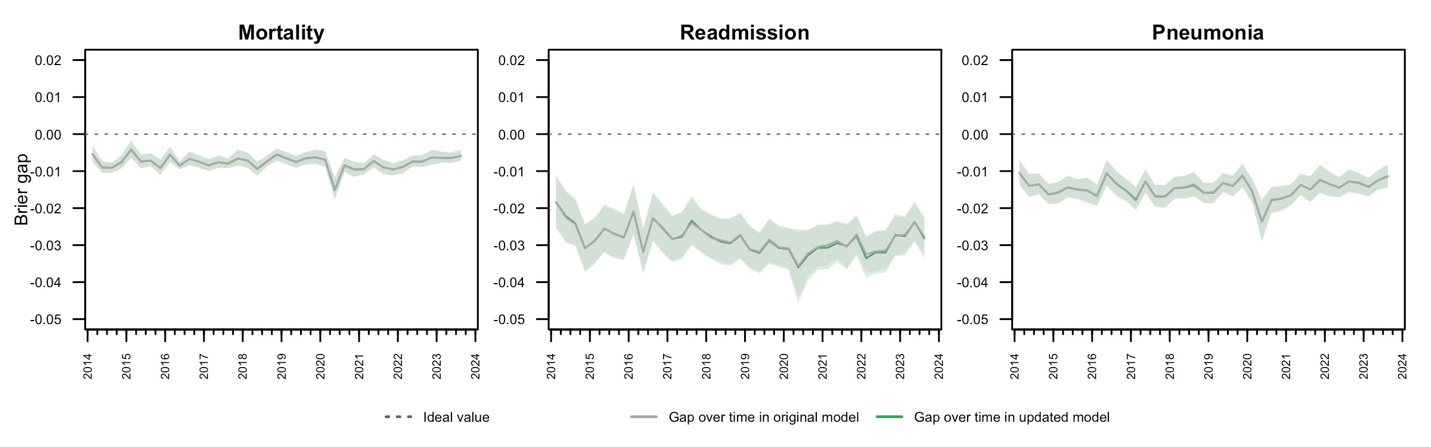

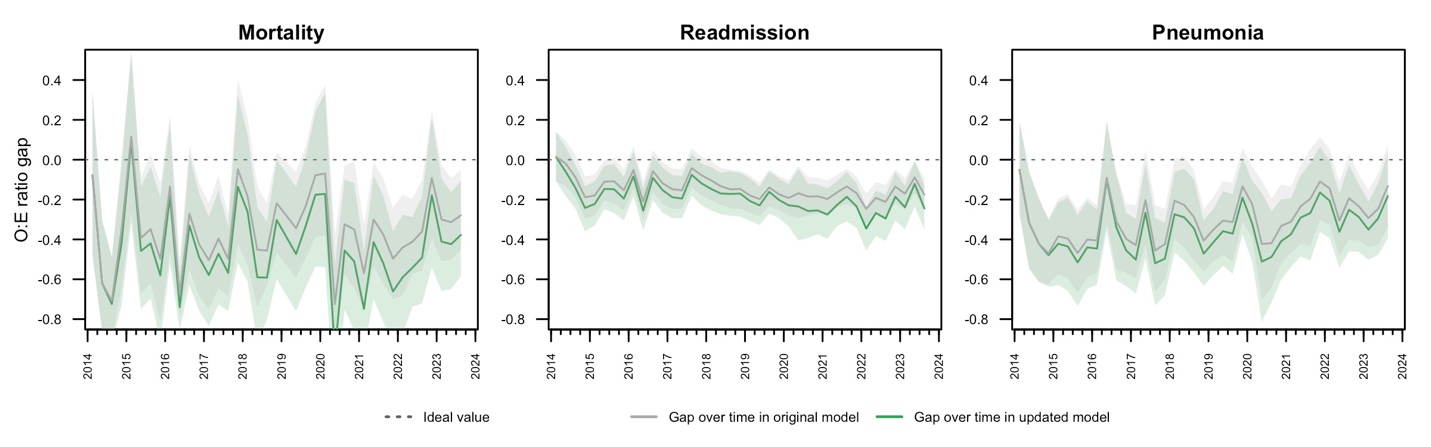

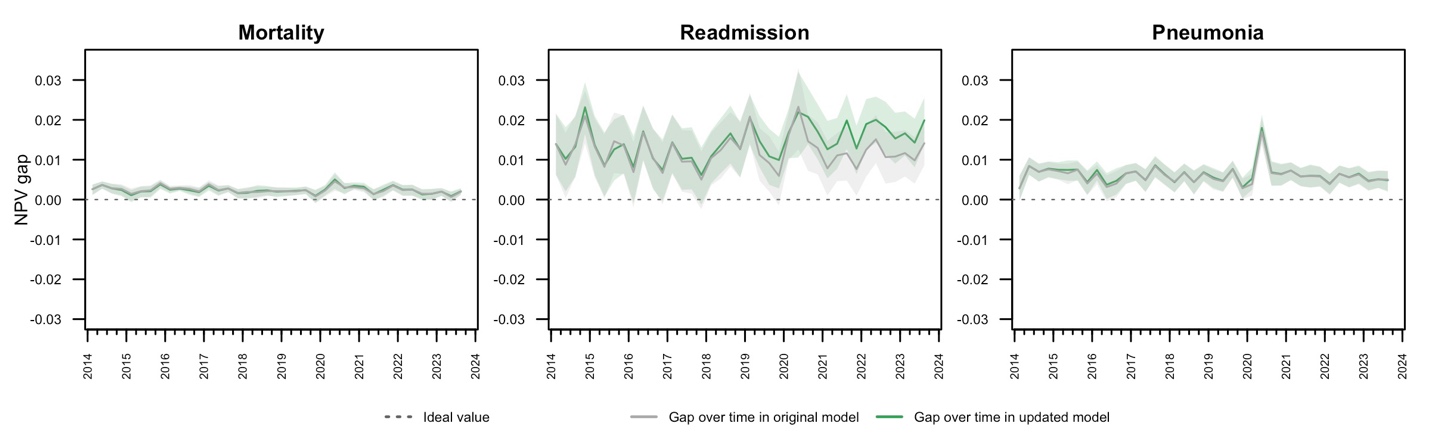

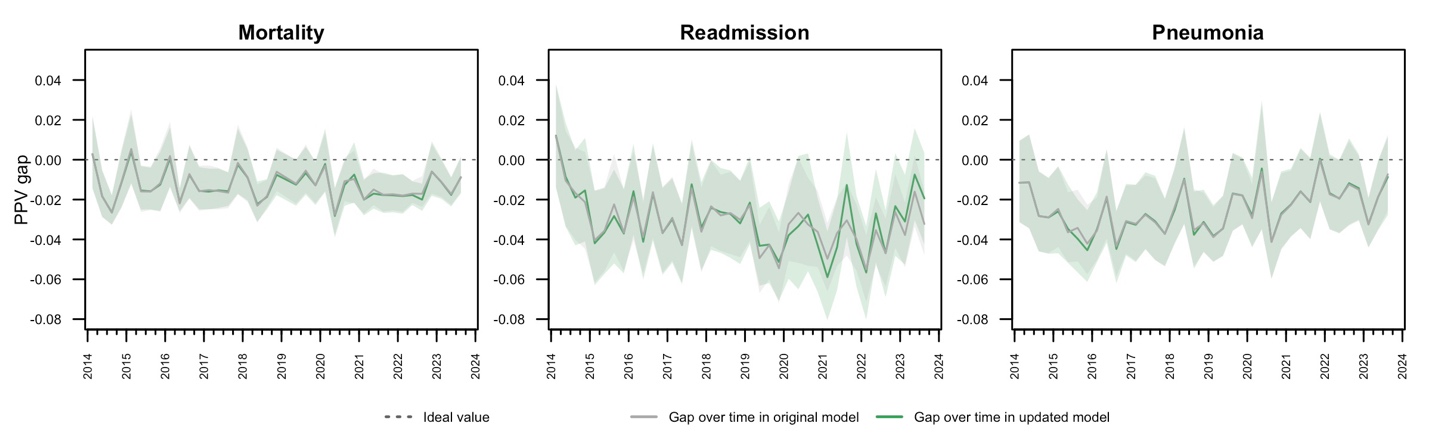

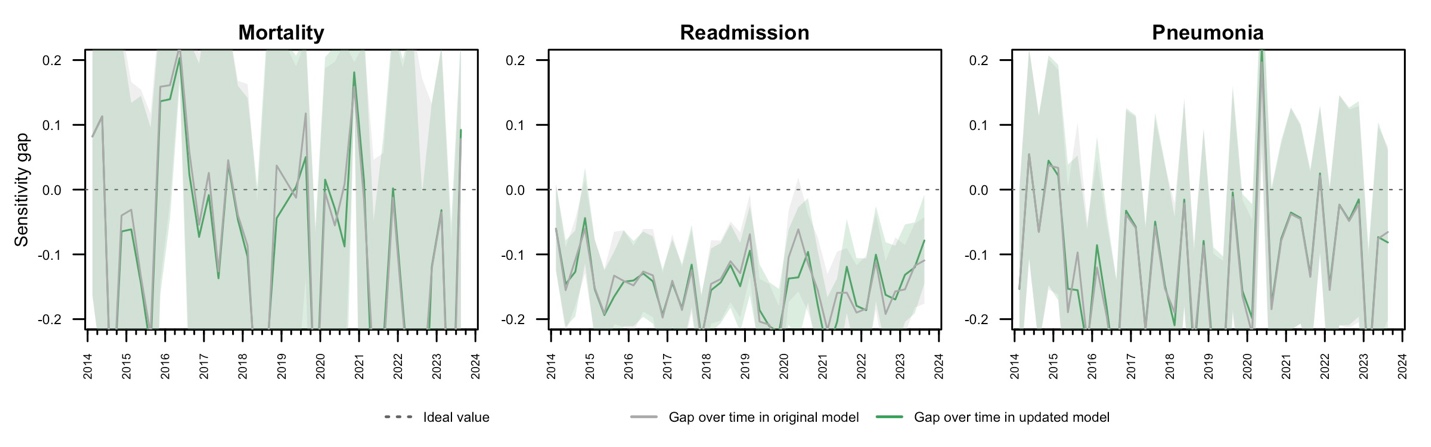

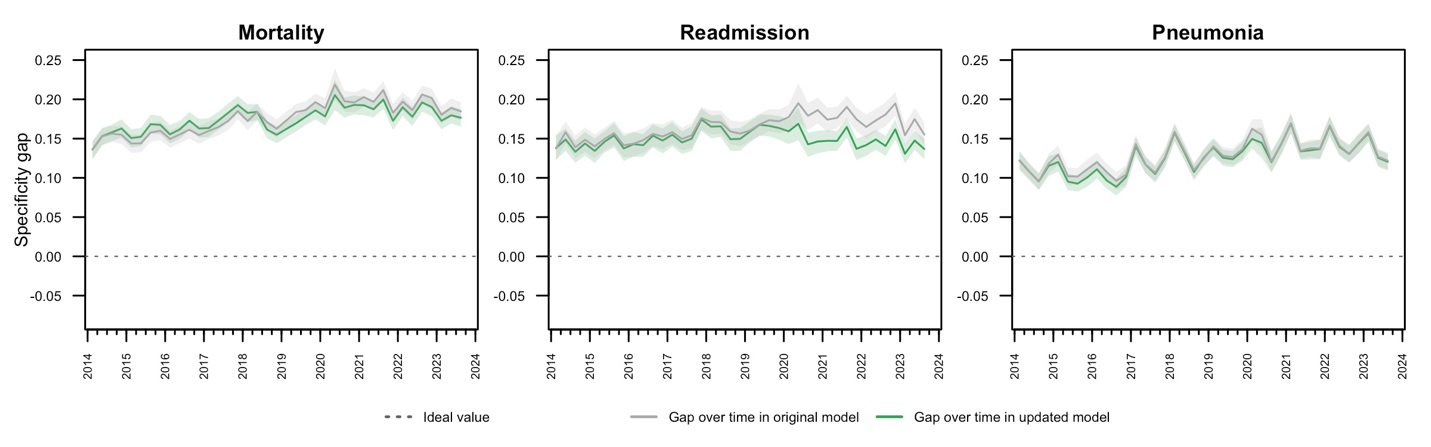

Supplement: ocaf039_Supplementary_Data [file ocaf039_supplementary_data.docx]
